# Genome-Scale Transcription-Translation Mapping Reveals Features of Zymomonas mobilis Transcription Units and Promoters

**DOI:** 10.1128/mSystems.00250-20

**Published:** 2020-07-21

**Authors:** Jessica M. Vera, Indro Neil Ghosh, Yaoping Zhang, Alex S. Hebert, Joshua J. Coon, Robert Landick

**Affiliations:** a Great Lakes Bioenergy Research Center, University of Wisconsin—Madison, Madison, Wisconsin, USA; b Genome Center of Wisconsin, University of Wisconsin—Madison, Madison, Wisconsin, USA; c Department of Chemistry, University of Wisconsin—Madison, Madison, Wisconsin, USA; d Department of Biomolecular Chemistry, University of Wisconsin—Madison, Madison, Wisconsin, USA; e Department of Biochemistry, University of Wisconsin—Madison, Madison, Wisconsin, USA; f Department of Bacteriology, University of Wisconsin—Madison, Madison, Wisconsin, USA; g Cell and Molecular Biology Graduate Training Program, University of Wisconsin—Madison, Madison, Wisconsin, USA; University of California, Berkeley

**Keywords:** *Zymomonas mobilis*, genome annotation, proteogenomics, transcription start site sequencing, promoter elements

## Abstract

Zymomonas mobilis is an ethanologenic alphaproteobacterium with promise for the industrial conversion of renewable plant biomass into fuels and chemical bioproducts. Limited functional annotation of the Z. mobilis genome is a current barrier to both fundamental studies of Z. mobilis and its development as a synthetic biology chassis. To gain insight, we collected sample-matched multiomics data, including RNA sequencing (RNA-seq), transcription start site (TSS) sequencing (TSS-seq), termination sequencing (term-seq), ribosome profiling, and label-free shotgun proteomic mass spectrometry, across different growth conditions and used these data to improve annotation and assign functional sites in the Z. mobilis genome. Proteomics and ribosome profiling informed revisions of protein-coding genes, which included 44 start codon changes and 42 added proteins. We developed statistical methods for annotating transcript 5′ and 3′ ends, enabling the identification of 3,940 TSSs and their corresponding promoters and 2,091 transcription termination sites, which were distinguished from RNA processing sites by the lack of an adjacent RNA 5′ end. Our results revealed that Z. mobilis σ^A^ −35 and −10 promoter elements closely resemble canonical Escherichia coli −35 and −10 elements, with one notable exception: the Z. mobilis −10 element lacks the highly conserved −7 thymine observed in E. coli and other previously characterized σ^A^ promoters. The σ^A^ promoters of another alphaproteobacterium, Caulobacter crescentus, similarly lack the conservation of −7 thymine in their −10 elements. Our results anchor the development of Z. mobilis as a platform for synthetic biology and establish strategies for empirical genome annotation that can complement purely computational methods.

**IMPORTANCE** Efforts to rationally engineer synthetic pathways in Zymomonas mobilis are impeded by a lack of knowledge and tools for predictable and quantitative programming of gene regulation at the transcriptional, posttranscriptional, and posttranslational levels. With the detailed functional characterization of the Z. mobilis genome presented in this work, we provide crucial knowledge for the development of synthetic genetic parts tailored to Z. mobilis. This information is vital as researchers continue to develop Z. mobilis for synthetic biology applications. Our methods and statistical analyses also provide ways to rapidly advance the understanding of poorly characterized bacteria via empirical data that enable the experimental validation of sequence-based prediction for genome characterization and annotation.

## Abstract (precis)

Efforts to rationally engineer synthetic pathways in Zymomonas mobilis are impeded by a lack of knowledge and tools for predictable and quantitative programming of gene regulation at the transcriptional, posttranscriptional, and posttranslational levels. With the detailed functional characterization of the Z. mobilis genome presented in this work, we provide crucial knowledge for the development of synthetic genetic parts tailored to Z. mobilis. This information is vital as researchers continue to develop Z. mobilis for synthetic biology applications. Our methods and statistical analyses also provide ways to rapidly advance the understanding of poorly characterized bacteria via empirical data that enable the experimental validation of sequence-based prediction for genome characterization and annotation.

## INTRODUCTION

Rapid advances in next-generation sequencing have produced a wealth of sequenced bacterial genomes. These sequences encode multiple layers of information, but the value of these exponentially expanding sequence data is limited without accurate annotations of genomic transcription and translation programs. Computational predictions provide an important starting point for the genomic annotation of newly sequenced genomes, but limitations in the accurate detection of small genes, signal peptides, overlapping open reading frames, and transcriptional and translational start and stop sites remain problematic (1–3). New high-throughput, empirical annotation strategies, which can complement sequence-based predictions, are needed to keep pace with the explosion of bacterial genome sequences and to leverage this information for the study of the large number of nonmodel bacteria that play diverse and important roles but lack the benefit of decades of functional studies. To that end, we report an integrated, multiomics approach to empirical annotation applied to the alphaproteobacterium Zymomonas mobilis.

Z. mobilis is a facultative anaerobe and obligate ethanologen (4, 5) that holds great promise as a microbial platform for the conversion of plant biomass into renewable fuel and chemical bioproducts (6, 7). However, limited empirical annotation of the Z. mobilis genome remains a crucial barrier to both basic studies of Z. mobilis and its development for synthetic biology. Genome sequences for seven Z. mobilis subsp. *mobilis* strains have been deposited in GenBank, including the reference strain ZM4 (ATCC 31821), for which its single ∼2-Mb circular chromosome and four 32- to 40-kb plasmids were definitively updated in 2019, after the initial publication in 2005 and subsequent revision (8–10). As is the case for many nonmodel bacteria, there is no central community database for Z. mobilis and little to no organized effort to generate or leverage genome-scale empirical data for its curation. Both proteomic and transcriptomic analyses have been conducted on Z. mobilis and used to elucidate its responses to oxygen, stresses including ethanol, and alternative carbon sources at the protein or gene level (10–20). However, high-precision, genome-scale approaches that can define an organism’s transcription and translation start and stop sites have not yet been applied to Z. mobilis, including (i) high-resolution RNA sequencing (RNA-seq), which provides a global view of transcript expression and organization (21); (ii) transcription start site sequencing (TSS-seq) (22); and (iii) termination sequencing (term-seq), which targets transcript 3′ termini (23). Together, these methods provide precise transcript boundaries and are indispensable for characterizing alternative transcription programs and genomic regulatory sequences. Although versions of these methods are already in use, here we report improvements to these approaches as well as rigorous statistical methods that enable the robust detection of transcript boundaries from TSS-seq and term-seq data. A commercially available enzyme (RNA 5′-pyrophosphohydrolase [RppH]) was validated for TSS-seq, and the accuracy of transcription termination site (TTS) identification was improved by the detection and assignment of RNA processing sites using mapped RNA 5′ and 3′ termini. Methods also exist that can provide an in-depth characterization of an organism’s proteome, such as ribosome profiling (24, 25) and shotgun proteomic mass spectrometry (26, 27).

We applied these techniques to Z. mobilis ZM4 grown under three different conditions, rich medium with and without O_2_ and minimal medium without O_2_, to generate a comprehensive, precise, and empirically refined annotation of the Z. mobilis genome. These results not only established methodological strategies to empirically expand bacterial genome annotation that exceed the capabilities of sequence-based annotation prediction tools but also yielded surprising new insight into the consensus sequence for the major (σ^A^) class of Z. mobilis promoters.

## RESULTS

### Matched multiomics samples were collected in exponential and stationary phases with and without O_2_.

**FIG 1:**
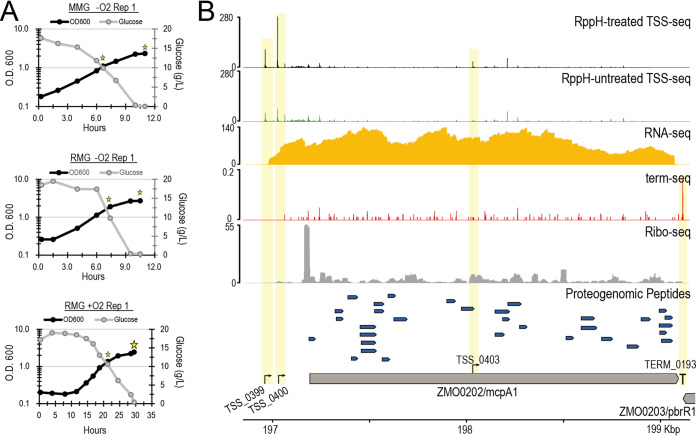
Matching multiomics samples collected during batch fermentations. (A) Single-replicate, representative cellular growth curves (via the OD_600_) (black lines) and medium glucose concentrations (gray lines) during batch fermentations of Z. mobilis ZM4 grown in minimal medium with glucose (MMG) anaerobically (−O_2_), rich medium with glucose (RMG) anaerobically, and RMG aerobically (+O_2_). Yellow stars mark the time points at which multiomics samples were collected. (B) Example of multiomics data from cells grown anaerobically in MMG at the mid-glucose-phase time point for the ZMO0202 locus where two upstream TSSs, one intragenic TSS, and one TTS were identified. Tracks from top to bottom are TSS-seq normalized read coverage (black and green), RNA-seq read coverage (gold), term-seq normalized read coverage (red), ribosome-profiling read coverage (gray), proteogenomic peptide identification (blue), and genome annotations. Data were visualized using pyGenomeTracks (71).

We grew Z. mobilis ZM4 under three different conditions: rich medium with glucose (RMG) anaerobically, RMG aerobically, and minimal medium with glucose (MMG) anaerobically. Z. mobilis grew poorly in MMG aerobically. Both the cell density (apparent optical density at 600 nm [OD_600_]) and the extracellular glucose concentration were monitored during the cultivations (Fig. 1A; see also Fig. S1 in the supplemental material). To examine the transcriptome, translatome, and proteome of Z. mobilis, we collected cells for RNA isolation, ribosome profiling (ribo-seq), and proteomics at two time points from each culture: a growth-phase time point (sampled when 50% of glucose remained in the medium) and a stationary-phase time point (sampled 1 h after glucose depletion) (Fig. 1A, yellow stars). Three (MMG and RMG anaerobic) or four (RMG aerobic) biological-replicate cultivations were performed; only the stationary-phase sample was obtained for the fourth RMG aerobic replicate, making 19 samples total. From the multiomics samples collected, we generated data using RNA-seq, TSS-seq (22), term-seq (23), ribo-seq (24, 25), and label-free shotgun proteomics by liquid chromatography-tandem mass spectrometry (LC-MS/MS). Each of these genome-scale data sets was used to empirically refine or identify genomic features in Z. mobilis ZM4 (illustrated for ZMO0202 [*mcpA1*]) (Fig. 1B).

10.1128/mSystems.00250-20.1 FIG S1 Growth and glucose consumption of all batch fermentations from this study. The cellular growth curve (monitored using the apparent OD_600_) (black line) and the remaining glucose concentrations (gray line) are shown for each of the biological replicates (three in anaerobic minimal medium with glucose [MMG] [top row], three in anaerobic rich medium with glucose [RMG] [middle row], and four in aerobic RMG [bottom row]). Yellow stars mark the time points at which multiomics samples were collected. In the fourth aerobic RMG replicate, only the stationary-phase sample was obtained, making the total number of samples 19. Download FIG S1, JPG file, 0.5 MB. Copyright © 2020 Vera et al. 2020 Vera et al. This content is distributed under the terms of the Creative Commons Attribution 4.0 International license.

Consistent with some previous reports, Z. mobilis grew more slowly aerobically in RMG than anaerobically in both RMG and MMG (15, 19). When grown anaerobically, cultures reached the stationary-phase time point within ∼10 h, whereas aerobic cultures required up to 30 h. This difference in growth between aerobic and anaerobic conditions stems from both a longer lag phase (∼10 h) and an increased doubling time in aerobic cultures (Fig. 1; Fig. S1).

### Genome annotation revisions aided by proteogenomics and ribosome profiling.

Accurate and complete gene annotations, particularly protein-coding gene annotations, are crucial for genome-scale and systems-level research in any organism and were a necessary predicate for our mapping of transcriptional signals. Thus, we first applied a proteogenomics analysis to the label-free shotgun proteomics data to comprehensively annotate protein-coding genes in Z. mobilis. Proteogenomics differs from standard quantitative proteomics by matching peptide spectra against a six-way translation of the target organism’s genome as opposed to searching against a database of established protein-coding gene annotations. We performed a proteogenomics search against a database of all amino acid sequences of ≥20 amino acids in length from a six-way translation of the ZM4 genome (in total, 65,246 sequences). We set search parameters that would identify both N- and C-terminal peptides, including formyl-Met N-terminal peptides and Val to Met or Leu to Met at any peptide N terminus to account for alternative start codon usage. We identified a total of 23,455 distinct peptides that were present in at least one sample, with 51% of peptides being identified in at least 15 out of 19 samples (Fig. S2).

10.1128/mSystems.00250-20.2 FIG S2 Sample distribution of proteogenomic peptides. (A) Histogram showing the sample distribution of the 23,455 distinct peptides identified by proteogenomics. Approximately 10% of peptides were identified in only 1 sample, while more than 30% of peptides were found in all 19 samples. Cumulatively, 51% of peptides could be found in 15 or more samples. (B) Bar graph showing the detection of peptides within a set of biological replicates. The *y* axis indicates the percentage of peptides detected in all biological replicates for that condition (the intersection) over all distinct peptides found in the replicates (the union). Download FIG S2, JPG file, 0.4 MB. Copyright © 2020 Vera et al. 2020 Vera et al. This content is distributed under the terms of the Creative Commons Attribution 4.0 International license.

About 96% of peptides identified corresponded to protein-coding gene annotations in Z. mobilis ZM4 in the 2019 GenBank records under accession numbers CP023715.1, CP023716.1, CP023717.1, CP023718.1, and CP023719.1. Recently, the NCBI computationally reannotated these ZM4 chromosome and plasmid sequences (10) using the Prokaryotic Genome Annotation Pipeline (PGAP) (3) (available under RefSeq accession numbers NZ_CP023715.1, NZ_CP023716.1, NZ_CP023717.1, NZ_CP023718.1, and NZ_CP023719.1). There were several differences in the PGAP annotations relative to previously reported ZM4 annotations, including 49 genes unique to PGAP, 28 genes unique to previous annotations, and 106 genes with differing starts, stops, or both. The majority of differences in gene annotations occurred at the 5′ ends of protein-coding genes, which highlights the challenges of computationally selecting a gene’s start codon when multiple in-frame start codons are present. Differences in stop codon coordinates all corresponded to pseudogenes.

We used both proteogenomics and ribo-seq data to examine differences in start codon assignments between PGAP and previous annotations (see Materials and Methods). Proteogenomic peptides supported the 5′ extension of 10 proteins, and ribo-seq supported another 6 (Table 1; Table S1). For 5 and 14 genes, proteogenomics and ribo-seq, respectively, supported the retention of start codon sites from previous annotations that were otherwise computationally predicted by PGAP to be shorter. Of the remaining genes with start codon differences, we selected the longest version of a protein-coding gene unless the shorter version had a methionine start codon and the longer version did not, which resulted in 4 shorter genes and 24 longer genes than in previous ZM4 annotations (Table 1; Table S1). It is important to note that multiple, in-frame start codons may contribute to alternative translation initiation events; however, we could not distinguish multiple translation initiation sites with our proteogenomics or ribosome profiling data. Thus, we used the longest protein product in these cases.

**TABLE 1:** Summary of Z. mobilis ZM4 genome annotation revisions^*a*^

Feature revision	No. of genes
Genes not changed	1,891
Predicted start change/longer gene	40
Predicted start change/shorter gene	4
New pseudogene assignments	11
Total pseudogenes	19
Genes reassigned sequence	1
Protein-coding genes added	42
Other genes added^*b*^	4
Genes removed	4
Hypothetical proteins→uncharacterized proteins^*c*^	155
16S rRNA changes	1

a All changes are incorporated in updated GenBank records under accession numbers CP023715.1, CP023716.1, CP023717.1, CP023718.1, and CP023719.1.

b One each for tRNA, transfer-messenger RNA (tmRNA), RNase P RNA, and signal recognition particle RNA.

c Protein experimentally validated by proteomics/proteogenomics.

10.1128/mSystems.00250-20.5 TABLE S1 List of revisions to Z. mobilis ZM4 gene annotations. Download Table S1, PDF file, 0.1 MB. Copyright © 2020 Vera et al. 2020 Vera et al. This content is distributed under the terms of the Creative Commons Attribution 4.0 International license.

For the sake of completeness, we choose to incorporate all genes uniquely identified by PGAP into our revised set of ZM4 gene annotations (Table 1; Table S1). Proteogenomic peptides confirmed 6 out of 41 PGAP-unique protein-coding genes, and ribo-seq data supported another 3 genes. Proteogenomic peptides also identified a previously unannotated gene on plasmid pZM36. This unannotated protein, assigned the locus tag pZM36x049, exhibits sequence similarity to the hypothetical protein under RefSeq accession number WP_012954675.1 from the Z. mobilis strain CP4 plasmid pZZM401. We tabulated all changes to the ZM4 chromosome and plasmid gene annotations (Table 1) as well as new genomic coordinates for all revised genes (Table S1).

### Precise transcription unit start sites were defined using TSS-seq.

RNA-seq is routinely used to quantify and compare gene expression levels, but it can also be used to identify novel transcripts, gene boundaries, transcription unit (TU) organization, and transcript 5′ and 3′ termini. However, transcript termini can be only indirectly inferred when using traditional RNA-seq methods (21, 28), which limits the precision of TSS and TTS identification. Furthermore, with the traditional RNA-seq alternative, intragenic TSSs and TTSs are difficult to distinguish due to overlapping RNA-seq read coverage. Knowing correct and precise transcript termini is crucial for defining DNA regulatory regions such as promoters, terminators, 5′ and 3′ untranslated regions (UTRs), small RNAs (sRNAs), and attenuation control elements.

TSS-seq is a high-precision sequencing method that directly identifies TSSs in bacteria by directly ligating a sequencing adapter to RNA 5′ ends. To distinguish TSSs from RNA processing sites in bacteria, TSS-seq selectively identifies 5′-triphosphoryl ends generated by transcription initiation. TSSs corresponding to 5′-triphosphoryl ends can be assigned by different methods, including (i) enrichment (e.g., enzymatic generation of a 5′ cap followed by cap affinity enrichment) (29), (ii) comparing ratios of reads from an adapter ligated to 5′-monophosphoryl RNAs before and after pretreatment with a 5′ exonuclease that selectively degrades monophosphoryl RNAs and the subsequent conversion of 5′-triphosphoryl to 5′-monophosphoryl RNAs (known as differential RNA-seq; dRNA-seq) (30), and (iii) comparing the ratios of reads from an adapter ligated to 5′-monophosphoryl RNAs before and after the conversion of 5′-triphosphoryl to 5′-monophosphoryl ends (e.g., by treatment with tobacco acid pyrophosphatase) (22). For this study, we chose a ratio approach that compared two sequencing libraries, one in which 5′-triphosphoryl RNAs were enzymatically converted to 5′-monophosphoryl RNAs alongside an untreated control library that reports the background of preexisting 5′-monophosphorylated RNAs.

**FIG 2:**
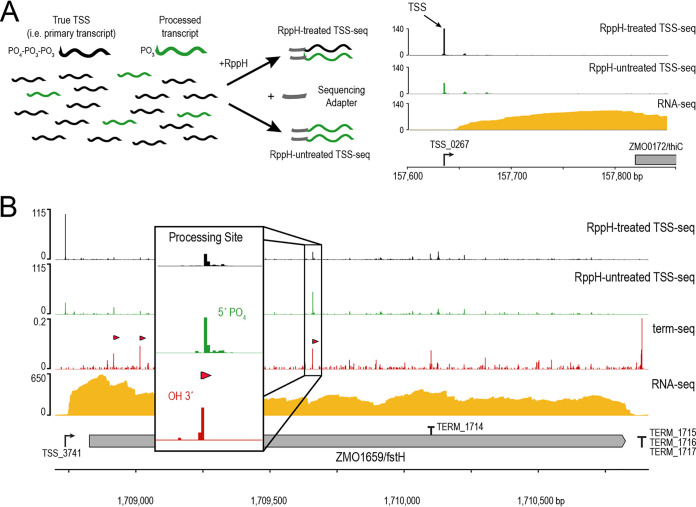
Identification of transcript termini by TSS-seq and term-seq. (A) Schematic of the TSS-seq library preparation strategy. True TSSs have a 5′-triphosphoryl moiety, while processed and degraded RNAs have a 5′-monophosphoryl moiety. RppH treatment is necessary to ligate a sequencing adapter to the 5′ end of true TSSs but results in the sequencing of both tri- and monophosphoryl RNAs. TSSs are identified as having greater read coverage in RppH-treated libraries in comparisons between RppH-treated and RppH-untreated RNA samples. The right panel is an example of a ZMO0172 (*thiC*) TSS identified ∼200 bp upstream of the ZMO0172 start codon. Tracks display RMG anaerobic mid-glucose-phase TSS-seq normalized read coverage and RNA-seq read coverage. (B) Schematic of processing site versus TTS identification via the integration of RppH-untreated TSS-seq (green) with term-seq (red) data within ZMO1659. RNA 5′-monophosphoryl termini were identified from RppH-untreated TSS-seq data, and these sites were used to distinguish between RNA 3′-hydroxyl termini pertaining to processing sites (marked with red triangles in the term-seq track) and RNA 3′-hydroxyl termini pertaining to TTSs.

Although tobacco acid pyrophosphatase has been the preferred enzyme for the conversion of 5′-triphosphates to 5′-monophosphates, this enzyme is no longer commercially available. Therefore, we tested Escherichia coli RNA 5′-pyrophosphohydrolase (RppH) as a replacement for tobacco acid pyrophosphatase using an *in vitro* assay in which we observed the conversion of a radiolabeled, 5′-triphosphoryl RNA to a monophosphoryl RNA over time (Fig. S3). Under our assay conditions, RppH gave complete conversion within 30 min, confirming that RppH was suitable for pretreating RNA for TSS-seq library construction. When RppH was used to pretreat samples for TSS-seq, true TSSs exhibited a sharp increase in read coverage relative to RppH-untreated samples, thereby providing an accurate and robust report of TSS locations (Fig. 2A). The experimentally validated, RppH-based, TSS-seq method was then applied to all RNA samples from the growth- and stationary-phase Z. mobilis RNA preparations.

10.1128/mSystems.00250-20.3 FIG S3 *In vitro* RppH activity assay. RppH pyrophosphohydrolase activity was assayed against a radiolabeled, *in vitro*-transcribed, 26-mer RNA (A26). A26 RNA was generated as a mix of both 5′-tri- and -monophosphoryl species, as indicated by the presence of two resolving bands labeled P-P-P-A26 and P-A26, respectively. A26 was mixed with 2.5 μg of Z. mobilis purified total RNA or used alone as a control and treated with RppH for 1/2 to 3 h total and for 0 h as a control. In the presence of RppH, the P-P-P-A26 and P-A26 bands collapse into a single band corresponding to P-A26 only, confirming RppH activity. Download FIG S3, JPG file, 0.6 MB. Copyright © 2020 Vera et al. 2020 Vera et al. This content is distributed under the terms of the Creative Commons Attribution 4.0 International license.

We applied an annotation-agnostic statistical analysis to identify TSSs from our TSS-seq data (see Materials and Methods). Previous studies utilizing TSS-seq have relied on subjective, static criteria for TSS identification; were often restricted to intergenic regions; and did not include statistical testing. Briefly, we identified TSSs using the DESeq2 differential gene expression analysis package to identify positions, genome wide, at which reads in RppH-pretreated libraries exceeded those in RppH-untreated (preexisting 5′-monophosphoryl RNAs) libraries with a false discovery rate (FDR) of <0.05. Our DESeq2 pipeline identified 4,652 positions as candidate TSSs under at least one growth condition. We were also able to identify some processing sites distinct from TSSs because they were 1 nucleotide (nt) after an RNA 3′ end identified during term-seq experiments (Fig. 2B), described in detail in the term-seq section below.

Under each condition, TSSs were further refined by first classifying primary and secondary sites, for which secondary sites were defined as TSSs immediately adjacent (i.e., no intervening nucleotides) to another TSS but with lower read coverage in the RppH-treated libraries. After removing all secondary TSSs, 3,940 positions were identified as TSSs under at least one condition (Table 2; Data Set S1). We note that secondary TSSs may reflect alternative initiating nucleotides at some promoters, which are known to occur due to flexibility in template DNA strand positioning and can be affected by *in vivo* nucleoside triphosphate (NTP) concentrations (31, 32).

**TABLE 2:** Summary of TSSs and TTSs identified under each condition

Medium	Time point	Presence of O_2_	Total no. of TSSs	No. of condition- specific TSSs	Total no. of TTSs	No. of condition- specific TTSs
MMG	Mid-glucose	−	2,248	948	692	179
MMG	Stationary	−	1,638	550	770	315
RMG	Mid-glucose	−	1,089	71	632	85
RMG	Stationary	−	1,343	304	501	65
RMG	Mid-glucose	+	1,143	17	956	17
RMG	Stationary	+	1,012	42	817	61

10.1128/mSystems.00250-20.6 DATA SET S1 Transcription start site (TSS) catalog. Sheet 1 shows column legends for sheets 2, 3, and 4. Sheet 2 shows TSSs mapped in the Z. mobilis ZM4 genome. Sheet 3 shows TSS validation by comparison to promoter regions reported previously by Y. Yang, W. Shen, J. Huang, R. Li, et al. (Biotechnol Biofuels 12:52, 2019, https://doi.org/10.1186/s13068-019-1399-6). Sheet 4 shows TSS validation by comparison to transcript 5′ ends reported previously by S. H. Cho, R. Lei, T. D. Henninger, and L. M. Contreras (Appl Environ Microbiol 80:4189–4198, 2014, https://doi.org/10.1128/AEM.00429-14) and S. H. Cho, K. Haning, W. Shen, C. Blome, et al. (Front Microbiol 8:2432, 2017, https://doi.org/10.3389/fmicb.2017.02432). Download Data Set S1, XLSX file, 0.6 MB. Copyright © 2020 Vera et al. 2020 Vera et al. This content is distributed under the terms of the Creative Commons Attribution 4.0 International license.

To validate our TSS mapping data, we compared them to two types of published promoter data for Z. mobilis: (i) promoter regions whose activity is verified by a reporter assay (33) and (ii) the 5′-end coordinates of 5′ UTRs or sRNAs that are mapped by rapid amplification of cDNA ends (RACE) (11, 12). All but 2 of 19 promoter regions with strong activity contained at least one and on average five TSSs (versus only five promoters with an average of one TSS for a randomized control; *P *= 0.0004) (Data Set S1). Of 47 RNA 5′ ends mapped by RACE, more than half contained a TSS within 20 bp with a median distance of 13 bp for the entire set of 47, versus a median distance of 673 bp for the randomized control (*P *< 0.0001) (Data Set S1). We conclude that our TSS mapping data strongly correlate with known promoters and TSSs in Z. mobilis.

### Alternative TSS usage contributes to transcriptome complexity in Z. mobilis.

**FIG 3:**
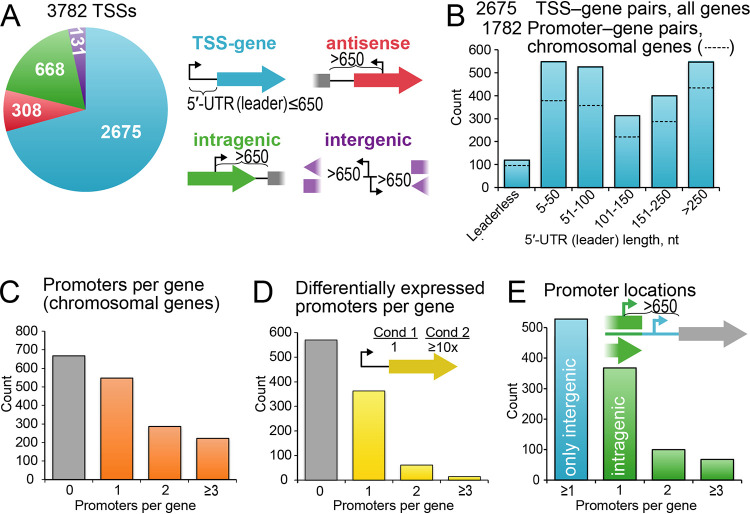
Characteristics of Z. mobilis TSSs and promoters. (A) Of 3,782 primary TSSs, most are associated with the expression of one or more genes (TSS-gene), but notable numbers of TSSs may be associated with the synthesis of noncoding RNAs at antisense, intragenic, or intergenic locations. (B) Although some TSS-gene pairs produce leaderless mRNAs, most produce mRNAs with 5′ UTRs (leaders) long enough to encode regulatory mechanisms (e.g., 1,397 UTRs of >100 bp versus 1,278 UTRs of ≤100 bp). Because some TSSs are within 10 nt of each other and plasmid TSSs may have different properties, we also considered coalesced TSSs to define likely chromosomal promoters (dotted lines), for which the same statement is true (1,108 UTRs of >100 bp versus 962 UTRs of ≤100 bp). (C) Most Z. mobilis genes are associated with one or more promoters (1,010 of 1,738 chromosomal genes). (D) A large fraction (0.44) of 1,010 Z. mobilis chromosomal genes associated with promoters are associated with one or more regulated promoters (a promoter with greater than the chromosomal mean promoter activity under one condition that is greater by a factor of 10 or more than its activity under another condition; *n* = 440). (E) More than half of the chromosomal genes associated with promoters (536 of 1,010) are associated with at least one promoter located within an upstream gene.

Identification of TSSs is crucial to understanding gene regulatory mechanisms because TSSs identify promoters, which are associated with activator and repressor sites, and also because they define 5′ UTRs that can encode attenuation, riboswitch, and translational control mechanisms. Using our revised ZM4 gene annotations, we assessed the genomic distribution of all primary TSSs relative to protein-coding gene annotations (Fig. 3). We assigned TSSs to the nearest downstream start codon with a maximum leader length of 650 bp. Intragenic TSSs 650 bp or less from a downstream gene were assigned to that gene. Following this scheme, a total of 2,675 TSS–protein-coding gene pairs were assigned, 119 of which define leaderless transcripts (a transcript that starts at the translation start codon, i.e., lacking a 5′ UTR, which we defined operationally as a leader of ≤5 nt) (Fig. 3A and B; Data Set S1). Thus, Z. mobilis contains significantly more leaderless transcripts than E. coli, which is reported to contain five or fewer leaderless transcripts (34, 35). However, Z. mobilis contains far fewer leaderless transcripts than some bacteria (e.g., Mycobacterium tuberculosis, for which 25% of transcripts are reportedly leaderless) (36).

Based on these TSS-gene pairs, the median length of 5′ UTRs was 114 nt, suggesting that a large fraction were long enough to encode substrates for RNA-based regulatory mechanisms. To reduce bias in this estimate from closely spaced TSSs or episomal promoters, we examined only chromosomal promoters and coalesced TSSs within 10 bp of each other to single TSSs assigned to the position with the highest TSS-seq read count. For these 1,782 promoters, the mean 5′-UTR length (111 nt) and distribution remained similar to those of the uncoalesced TSSs (Fig. 3B). About a third of genes (*n *= 513) were associated with more than one coalesced promoter (Fig. 3C). About half of the leaderless TUs (*n *= 52) were also associated with an alternative, leadered TU initiating from an upstream promoter. These multiple promoters per gene or operon greatly increase the complexity of the Z. mobilis transcriptome and introduce possible alternative modes of regulating the expression of these genes, consistent with the complex use of multiple promoters per gene or operon found in other bacteria like E. coli (37). Consistent with this complexity, about half (44%) of the genes associated with a promoter had one or more promoters that exhibited ≥10-fold changes in normalized TSS-seq read counts (i.e., regulation) under different growth conditions (Fig. 3D; Data Set S1).

For approximately 34% of promoter-gene pairs, the promoter was located within an upstream gene (Fig. 3E). This occurrence of promoter sequences within genes in Z. mobilis is consistent with the precedent of regulatory complexity in other bacteria (38) and highlights the importance of not limiting searches for regulatory DNA sequences to intergenic regions. Intragenic TSSs may program the transcription of intraoperon genes, providing greater flexibility of gene expression and increasing the overall complexity of the Z. mobilis transcriptome.

**FIG 4:**
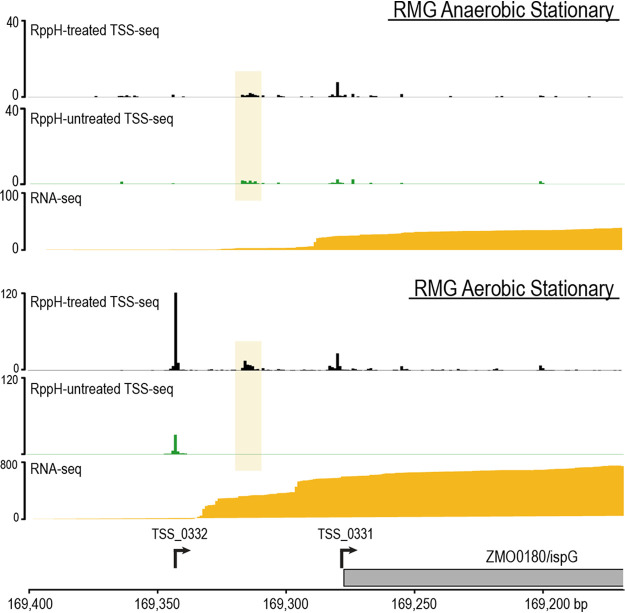
Condition-specific alternative TSS usage at *ispG*. Two TSSs were identified for ZMO0180 (*ispG*). TSS_0331 produces a leaderless *ispG* transcript, which was identified under all six tested conditions, while TSS_0332, which produces an *ispG* transcript with a 63-nt UTR, was identified only in aerobically grown samples. A third TSS is likely present within the yellow-shaded box but was not identified by our pipeline. The usage of the aerobic-specific TSS coincides with the upregulation of *ispG* at the stationary-phase time point (bottom three tracks) relative to anaerobically grown cells at the same time point (top three tracks). Differential TSS usage is further supported by RNA-seq read coverage (golden yellow), where coverage extends further upstream of the *ispG* coding region in aerobically grown samples. TSS-seq tracks (black and green) display condition-specific normalized read coverage.

To illustrate a specific example of regulatory complexity, we show the promoters for *ispG* (ZMO0180). This gene is of particular interest because *ispG* encodes a key oxygen-sensitive FeS enzyme required for isoprenoid synthesis and is transcribed either with a 63-nt 5′ UTR or as a leaderless transcript (Fig. 4). Interestingly, the −63 promoter appears to be favored when cells are grown under aerobic conditions; this TSS was identified only in aerobic samples, and an increase in RNA-seq coverage is apparent in the leader region for aerobic samples relative to anaerobic samples. Furthermore, *ispG* was found to be statistically differentially upregulated (FDR, <8.2 × 10^−5^) in stationary-phase aerobic samples relative to both anaerobic MMG and anaerobic RMG samples at the same time point, suggesting that the upregulation of *ispG* is dependent on the condition-specific usage of the −63 promoter. Based on our RNA-seq and TSS-seq data, a third *ispG* promoter may be located ∼38 bp before the start codon, but this position did not reach statistical significance in our analytical pipeline.

Of the 1,107 TSSs not assigned to a gene, more than half (*n *= 668) were located within a protein-coding gene but were more than 650 bp from the nearest downstream gene (intragenic TSSs) (Fig. 3A; Data Set S1). These TSSs may represent intragenic, alternative TUs, including those for noncoding RNAs. About one-quarter of TSSs (*n *= 308) were antisense TSSs within a protein-coding gene, and a smaller fraction (*n *= 131) were intergenic but more than 650 bp from the nearest downstream translation start codon (Fig. 3A; Data Set S1).

Of 15 experimentally validated Z. mobilis small RNAs (*Zm*sRNAs) (12), we identified 27 TSSs within 25 bp of the predicted start site of 9 *Zm*sRNAs (Data Set S1), including *Zm*s6 and *Zm*s4, which a recent report found to be upregulated by ethanol stress and to have a significant impact on ethanol tolerance and production in Z. mobilis (13). E. coli is thought to produce more than a thousand noncoding transcripts, some of which are functional as regulatory RNAs (37, 39). Thus, a significant fraction of the TSSs not assigned to genes may reflect additional noncoding transcription typical of bacterial genomes (40, 41).

Assigning TSSs to genes not only allows the characterization of transcript architecture but also provides promoters for use in molecular and synthetic biology applications. Using the 2,675 TSS-gene pairs that we identified (Fig. 3A; Data Set S1), we compiled a constant-expression promoter catalog from genes with consistent RNA-seq expression levels across all mid-glucose-phase samples. We generated a hand-curated set of 14 promoter sequences that spanned an ∼100-fold range in relatively constant expression of the downstream gene, were the single or predominant TSS for the gene, and were ≤200 nucleotides upstream of the gene (Table 3). This constant-expression promoter catalog expands the small but growing set of genetic parts enabling the use of the Z. mobilis chassis for synthetic biology (33, 42) and illustrates the power of multiomics data in identifying promoters.

**TABLE 3:** Z. mobilis constant-promoter catalog^*a*^

TSS	Position	Strand	5′ UTR length (nt)	Locus tag	Gene	Description	RNA-seq gene expression level^*b*^		
							M − O_2_	R − O_2_	R + O_2_
TSS_3435	1546230	–	28	ZMO1520		Conserved hypothetical protein	418	482	381
TSS_2169	980442	–	105	ZMO0963		TetR family transcriptional regulator	655	821	703
TSS_0437	220931	+	180	ZMO0226	*sdh*	Short-chain dehydrogenase/reductase	1,076	1,106	1,043
TSS_1434	617212	+	173	ZMO0619	*flgA*	Flagellum basal body P-ring formation protein	1,331	1,215	1,564
TSS_1798	783747	–	0	ZMO0784	*gatC*	Glutamyl-tRNA(Gln) amidotransferase C subunit	1,261	1,257	1,050
TSS_3191	1420891	–	42	ZMO1406		Alpha/beta hydrolase fold protein	1,192	1,270	1,288
TSS_3157	1398315	+	0	ZMO1384	*era*	GTP-binding protein	2,368	2,383	2,277
TSS_3290	1489122	+	0	ZMO1467		ABC-2-type transporter	2,497	2,407	3,045
TSS_3396	1531155	+	0	ZMO1504		DUF1321 domain-containing protein	2,902	2,551	3,093
TSS_1713	740401	+	27	ZMO0738	*thiG*	Thiazole biosynthesis protein	3,806	4,307	3,870
TSS_3522	1588943	+	0	ZMO1556	*gshA*	Glutamate-cysteine ligase	3,646	4,394	4,837
TSS_2374	1067836	+	81	ZMO1052	*purC*	Phosphoribosylaminoimidazole- succinocarboxamide synthase	4,868	5,365	5,980
TSS_0557	285800	+	97	ZMO0279		Putative cold shock DNA-binding protein	21,012	24,456	21,417
TSS_2422	1014593	+	33	ZMO0997	*eda*	Dehydro-phospho-deoxygluconate aldolase/hydroxy-oxoglutarate aldolase	38,816	45,442	37,095

a Promoters exhibiting constant expression levels across different growth conditions and ranging in strength over an ∼100-fold range are shown.

b Gene expression levels from replicate means of DESeq normalized gene read counts (see Materials and Methods). M − O_2_, growth-phase gene expression in anaerobic minimal medium with glucose; R − O_2_, growth-phase gene expression in anaerobic rich medium with glucose; R + O_2_, growth-phase gene expression in aerobic rich medium with glucose.

### Identification of Z. mobilis promoters reveals a noncanonical *−*10 element.

The precise TSSs obtained from TSS-seq make it possible to accurately identify the promoter sequences responsible for initiating transcription in Z. mobilis. From the 3,940 distinct TSSs identified by our TSS-seq method, we sought to characterize σ^A^ promoter sequences in Z. mobilis by motif analysis (σ^A^ is the so-called housekeeping σ factor responsible for most transcription initiation in bacteria and is an ortholog of E. coli σ^70^). To find σ^A^ promoter elements, we used an information theory-based approach first described by Shultzaberger and colleagues, which was used to derive σ^70^ promoter elements in E. coli (43). The application of this method to Z. mobilis TSSs required two assumptions: (i) σ^A^ is responsible for the majority of transcription initiation events in Z. mobilis, and (ii) like E. coli σ^70^, Z. mobilis σ^A^ will recognize two hexamer sequences approximately −35 and −10 nucleotides upstream of the TSS that are separated by a spacer region of variable length. We applied this flexible σ factor-binding model to all 3,940 primary TSSs that we identified in Z. mobilis. Combining all TSSs regardless of the sample conditions and time points provided more potential promoters for identification, thus giving us the most comprehensive assessment of Z. mobilis σ^A^ promoter elements. As a control, we applied the same model to E. coli promoter sequences using the 2,672 primary TSSs identified for at least one condition or time point by dRNA-seq (37). Prior to analysis, both Z. mobilis and E. coli TSSs were refined by removing sites within 15 bases of another upstream TSS in the same orientation using the criteria described by Shultzaberger et al. (43), resulting in final sets of 3,080 distinct Z. mobilis promoters and 2,666 distinct E. coli promoters. Because our flexible model was specific for the detection of σ^70^/σ^A^-like promoter elements, there was no need to further refine the set of TSSs; promoters that did not conform to the model were dropped during the analysis.

**FIG 5:**
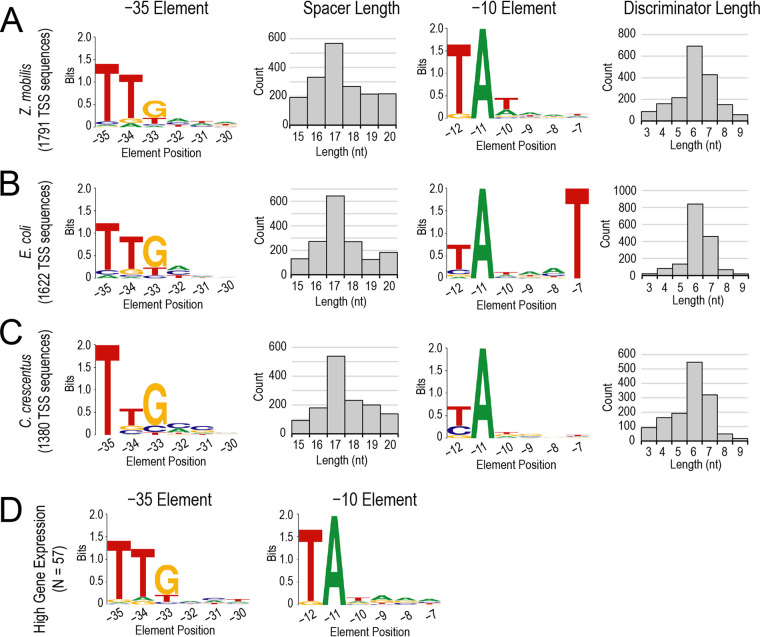
Novel sequence feature of the Z. mobilis σ^A^ −10 promoter element. (A to C) We applied a flexible model of σ^70^ promoters, first described by Shultzaberger et al. (43), to obtain σ^70^ −35 and −10 promoter elements for Z. mobilis (A), E. coli (B), and C. crescentus (C). All promoter element logos were generated with the WebLogo server (67). For each model, a histogram of discriminator and spacer lengths is also shown (gray-bar plots). Both Z. mobilis and C. crescentus were found to lack the conservation of thymine, or any other base, at position −7 of the −10 element, which is otherwise highly conserved in E. coli. (D) −10 and −35 element logos derived from highly expressed genes in Z. mobilis (*n* = 57).

Our promoter analysis identified 1,791 sequences that contributed to a Z. mobilis σ^A^ model of −35 and −10 elements with consensus sequences of TTGNNN and TANNNN, respectively (Fig. 5A; Data Set S2). The most prevalent discriminator length was 6 bp (“discriminator” is used here to indicate the sequence between the TSS and the −10 hexamer), and the most prevalent spacer length was 17 bp (sequence between −35 and −10 hexamers). Both the Z. mobilis and E. coli σ^A^/σ^70^ models yielded nearly identical consensus sequences, including similar discriminator and spacer length distributions, with one notable exception: the Z. mobilis −10 element lacks the highly conserved T_−7_ observed in E. coli (Fig. 5B; Data Set S2). Furthermore, no base was found to be highly conserved at position −7 in Z. mobilis. To interpret our findings and determine if the lack of base conservation at position −7 is specific to Z. mobilis, we also applied our σ^70^ model to Caulobacter crescentus promoter sequences identified by Zhou et al. (44) (Fig. 5C; Data Set S2). We found that like Z. mobilis, the C. crescentus −10 element also lacks a T at position −7 and exhibits no sequence conservation at this position. Thus, this divergence from the E. coli consensus −10 element is not specific to Z. mobilis but is also present in another alphaproteobacterium.

10.1128/mSystems.00250-20.7 DATA SET S2 σ^A^ promoter models. Sheet 1 shows Z. mobilis models. Sheet 2 shows E. coli models. Sheet 3 shows C. crescentus models. Download Data Set S2, XLSX file, 0.4 MB. Copyright © 2020 Vera et al. 2020 Vera et al. This content is distributed under the terms of the Creative Commons Attribution 4.0 International license.

Given the nearly indispensable nature of the T_−7_ in E. coli σ^70^ −10 elements (45–47), we also investigated whether specific promoter features (i.e., sequence, spacer, or discriminator length) correlated with higher levels of gene expression in Z. mobilis. Using RNA-seq gene counts from our MMG mid-glucose-phase samples, we examined all genes within the 75th to 90th percentiles of expression (263 total genes). We cross-referenced this set of highly expressed genes with the TSS-gene pairs, keeping only those genes with a single assigned TSS that had been identified in the MMG mid-glucose-phase samples. This yielded a subset of 57 promoter sequences for highly expressed genes (marked with * in Data Set S2), for which we observed a −10 consensus essentially identical to the consensus derived from all 1,791 promoters in our model (Fig. 5D). Thus, highly expressed genes in Z. mobilis are not dependent on a T_−7_ or any particular base at this position, in contrast to E. coli promoters, for which T_−7_ contributes greatly to promoter strength (48). We also observed no significant differences in the distribution of spacer and discriminator lengths in this subset of highly expressed genes. We conclude that the Z. mobilis σ^A^ promoter consensus, although similar to the well-known features found in model bacteria like E. coli and Bacillus subtilis, differs in the crucial −10 promoter element.

### Transcription termination sites were distinguished from processing sites via integration of term-seq and TSS-seq data.

Like TSS-seq, term-seq directly and selectively reports transcript 3′ termini based on the ligation of a sequencing adapter to RNA 3′-hydroxyls, which arise from both transcription termination and RNA processing. Term-seq was performed on all growth- and stationary-phase Z. mobilis RNA preparations. To assign RNA 3′ ends and, thus, candidate TTSs, we developed a statistical, annotation-agnostic method to identify TTSs using the Poisson test to identify positions, genome wide, with a high read count relative to a Poisson distribution built from a dynamic lambda parameter. This Poisson-based test was applied to each sample, and only sites with an FDR of <0.05 and that were found in at least two biological replicates were considered preliminary 3′ termini.

Although term-seq does not distinguish between 3′-transcript termini arising from transcription termination and those arising from RNA processing and degradation, we reasoned that many processing sites should yield a 3′-hydroxyl RNA (detected by term-seq), followed by a 5′-monophosphoryl RNA in the downstream position (Fig. 2B). Thus, we leveraged our RppH-untreated TSS-seq data to identify 5′-monophosphoryl sites using the same Poisson-based method for 3′-terminus identification and then integrated these results with preliminary 3′ sites to classify 3′ termini as processing sites or as TTSs. After eliminating 3′ termini likely arising from apparent processing or degradation by this criterion (1,954 total positions), a total of 2,091 positions were identified as candidate TTSs under at least one growth condition (Table 2; Data Set S3).

10.1128/mSystems.00250-20.8 DATA SET S3 Termination and RNA processing sites in Z. mobilis. Sheet 1 shows column legends for sheets 2, 3, and 4. Sheet 2 shows RNA processing sites. Sheet 3 shows transcription termination sites (TTSs). Sheet 4 shows matches between TTSs and terminators predicted by TransTermHP. Sheet 5 shows transcription terminators predicted by TransTermHP (C. L. Kingsford, K. Ayanbule, and S. L. Salzberg, Genome Biol 8:R22, 2007, https://doi.org/10.1186/gb-2007-8-2-r22). Download Data Set S3, XLSX file, 0.4 MB. Copyright © 2020 Vera et al. 2020 Vera et al. This content is distributed under the terms of the Creative Commons Attribution 4.0 International license.

### One-third of Z. mobilis TTSs appeared to result from intrinsic termination.

**FIG 6:**
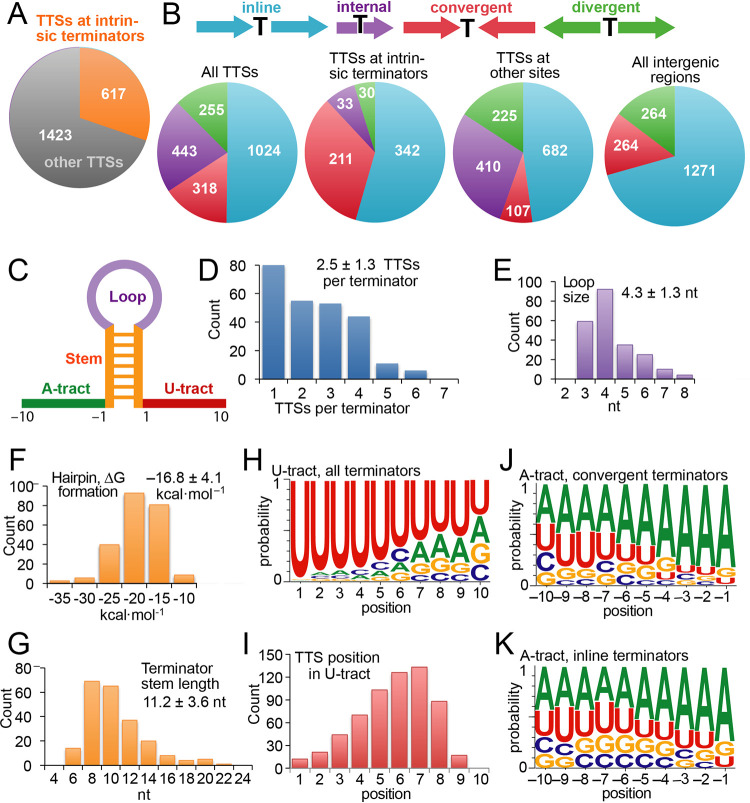
Properties of intrinsic terminators in Z. mobilis. (A) Number of TTSs that map to intrinsic terminators predicted in the Z. mobilis genome. (B) Locations of TTSs relative to the orientation of genes. Four general classes of terminator orientations are shown across the top: terminators between in-line genes (cyan), terminators within genes (purple), terminators between convergent genes (red), and terminators between divergent genes (green). For the first two classes, subclasses of terminator orientation are possible and are given in Data Set S3 in the supplemental material. Distributions of TTSs are shown in pie charts (left to right) for all TTSs, TTSs that mapped to predicted intrinsic terminators, and TTSs that did not map to predicted terminators. The pie chart on the right shows the distribution of all intergenic regions in Z. mobilis, for which the internal class is not applicable. (C) Color-coded diagram of intrinsic terminator parts. (D) Number of TTSs mapped per predicted intrinsic terminator. (E) Loop sizes of predicted intrinsic terminators to which TTSs were mapped. (F) Predicted free energy of formation (Δ*G*) of predicted intrinsic terminators to which TTSs were mapped. (G) Terminator hairpin stem lengths of predicted intrinsic terminators to which TTSs were mapped. (H) Sequence logo of the 249 predicted intrinsic terminators to which TTSs were mapped. (I) Positions of the TTSs in the U tract of predicted intrinsic terminators. (J) Sequence logo of the A tract (5′-flanking sequence) for predicted intrinsic terminators between convergent genes and to which TTSs were mapped. (K) Sequence logo of the A tract (5′-flanking sequence) for predicted intrinsic terminators between in-line genes and to which TTSs were mapped.

Transcription in bacteria is usually terminated by the ρ termination factor or by intrinsic terminators consisting of a nascent RNA hairpin followed by 7 to 9 nt of U-rich RNA (49), but the relative contributions of ρ-dependent and intrinsic termination vary among bacterial lineages. To ask what fraction of transcription termination in Z. mobilis occurs at intrinsic terminators, we predicted the locations of intrinsic terminators using TransTermHP (50). Consistent with the fraction of intrinsic termination observed in E. coli (51), we found that about one-third of chromosomal TTSs mapped to the U tracts of predicted intrinsic terminators (Fig. 6A). We examined the locations of these 249 experimentally validated intrinsic terminators (Data Set S3) by sorting the TTSs that mapped to the terminators and TTSs that mapped elsewhere into four classes of orientations relative to Z. mobilis genes: (i) terminators in line with adjacent genes, (ii) terminators internal to coding regions (e.g., >50 bp after an in-line AUG), (iii) terminators between convergent genes, and (iv) terminators between divergent genes (Fig. 6B). Consistent with the finding that E. coli intrinsic terminators often function bidirectionally between convergent genes (51), we found that the convergent class was overrepresented in TTSs mapping to intrinsic terminators relative to those mapping elsewhere (34% of matching TTSs versus only 7.5% of nonmatching TTSs). This overrepresentation is also notable because only 15% of intergenic regions in Z. mobilis are between converging genes (Fig. 6B). This result suggests that Z. mobilis may rely on positive supercoiling generated by opposing transcription units to enhance intrinsic termination between converging transcription units (51, 52).

We also used the experimentally verified intrinsic terminators to characterize the general properties of terminator hairpins and the flanking U and A tracts in Z. mobilis (Fig. 6C), finding mean hairpin stem and loop lengths of ∼11 bp and ∼4 nt, respectively, and an average predicted free energy of formation of ca. −17 kcal · mol^−1^ (Fig. 6E to G). These values are close to those reported for E. coli (51), consistent with a conserved mechanism of intrinsic termination between E. coli and Z. mobilis. TTSs were distributed throughout the U tract, with the most prominent positions being 7 nt after the terminator hairpin (Fig. 6H and I). Intrinsic termination is thought to occur in a window of 7 to 9 nt after the hairpin, so these data suggest some exonucleolytic trimming of intrinsically terminated RNAs in Z. mobilis. Adenosine residues were enriched upstream from intrinsic terminators located between convergent genes, as expected for a bidirectional terminator where the A’s correspond to a U tract on the opposite strand (Fig. 6J). Interestingly, however, A residues were also significantly enriched upstream from terminators found between in-line genes where a bidirectional function would not be expected (Fig. 6K). This finding of A tracts before terminators between in-line genes is consistent with a hypothesis that the A tract may also function to aid termination in the sense direction (53, 54).

## DISCUSSION

Z. mobilis has considerable potential as a synthetic biology chassis for the synthesis of plant biomass-derived bioproducts due to its stress tolerance and high-flux central C metabolism, but understanding its genomic organization, transcriptional and translational signals, and regulation is a prerequisite to realizing this potential. In this report, we describe the development and application of several new pipelines to exploit RNA-seq, TSS-seq, term-seq, ribosome-profiling, and proteogenomic data to progress toward this goal. These pipelines include new statistically robust ways to assign TSSs, RNA processing sites, and TTSs. In addition to providing an improved annotation of the Z. mobilis ZM4 genome and catalogs of promoters (TSSs) and terminators (TTSs), our key findings are that (i) even though the evolution of the simplified metabolism of Z. mobilis has yielded a small genome (∼2 Mb), the complexity evident in the transcriptional organization of larger bacterial genomes (multiple promoters per gene or operon, internal promoters in operons, and antisense and noncoding transcripts) holds true for Z. mobilis; (ii) unlike previously characterized bacterial promoters, Z. mobilis housekeeping (σ^A^) promoters do not utilize a conserved T_−7_ in the −10 promoter element to enable high levels of transcription initiation; and (iii) transcription termination signals in Z. mobilis, most notably intrinsic termination signals, appear to be similar to those of the model gammaproteobacterium E. coli. We discuss here each of these key findings and their implications.

The relatively simple lifestyle of Z. mobilis, which grows naturally only by the fermentation of glucose, fructose, or sucrose to ethanol, has allowed the evolution of a single 2-Mb genome accompanied by several small (<50-kb) plasmids. Nonetheless, our results reinforce the view that Z. mobilis exhibits transcriptional complexity similar to those of more extensively studied, more complex bacterial transcriptomes that are replete with nested operon architectures, small and noncoding RNAs, and riboregulators (23, 30, 51, 55, 56). In particular, the existence of large numbers of intragenic and antisense TSSs suggests that Z. mobilis expresses noncoding RNAs from TUs with genomic densities similar to those of E. coli and B. subtilis. These results are consistent with findings that Z. mobilis uses sRNA-based regulation to manage ethanol-induced and other stress responses (12–14). More broadly, however, the function of pervasive noncoding transcription in bacteria remains uncertain (40, 41); our results suggest that, in addition to regulatory sRNAs, pervasive noncoding transcription may have functions in Z. mobilis that remain to be discovered. Unlike E. coli but not uncommon in bacteria, including alphaproteobacteria like C. crescentus (57), Z. mobilis appears to produce many leaderless transcripts. Z. mobilis leaderless transcripts include those whose promoters produce relatively constant expression (Table 3), highlighting their possible use for the development of Z. mobilis synthetic biology parts.

In expanding the number of empirically defined TSSs and cognate promoters in Z. mobilis from a few (33, 42, 58) to more than a thousand (see Data Set S1 in the supplemental material) and applying sequence analyses, our results revealed the surprising lack of conservation of T_−7_ in the −10 element of housekeeping promoters in Z. mobilis as well as, in retrospect, C. crescentus (Fig. 5). Even strong Z. mobilis promoters exhibited no preference for T_−7_, begging the question of whether this corresponding binding site for T_−7_ in housekeeping σ^70^/σ^A^ is conserved in alphaproteobacteria. To investigate this question, we performed multiple-sequence alignment of σ^70^/σ^A^ regions 1.2 and 2 from Z. mobilis, C. crescentus, E. coli, Thermus aquaticus, B. subtilis, and *Mycobacterium tuberculosis* (Fig. S4). To stabilize the melting of promoter DNA, bacterial σ factors are thought to capture the nontemplate strand A_−11_ followed by the T_−7_ in two deep pockets formed by regions 1.2 and 2 (48). The T_−7_ pocket includes direct or water-mediated recognition contacts to the base by highly conserved L110, L111, and E114 in region 1.2 and N383, L386, K426, and S428 in σ region 2 (E. coli σ^70^ numbering). All seven of these residues are conserved in both Z. mobilis and C. crescentus σ^A^, suggesting that these alphaproteobacterial σ factors should be capable of selectively recognizing T_−7_, even though it does not appear to play important roles in promoter strength *in vivo*. This finding provides crucial guidance for the development of promoters for synthetic biology applications in Z. mobilis. Further work will be required to understand what role, if any, the T_−7_ contact plays in Z. mobilis transcriptional regulation.

10.1128/mSystems.00250-20.4 FIG S4 σ^70^/σ^A^ T_−7_-binding pocket residues are highly conserved. A multiple-sequence alignment of σ^70^/σ^A^ R1.2 and R2 protein domains from Z. mobilis, C. crescentus, *T. aquaticus*, B. subtilis, and M. tuberculosis σ^A^ and E. coli σ^70^ is shown. Based on the X-ray crystallographic structure of a *T. aquaticus* σ^A^ region-2–3 structural domain fragment bound to promoter DNA, Feklistov and Darst (48) identified 7 amino acids (indicated by boldface type) that participate in direct or water-mediated hydrogen bonds or van der Waals interactions with T_−7_. Download FIG S4, JPG file, 0.7 MB. Copyright © 2020 Vera et al. 2020 Vera et al. This content is distributed under the terms of the Creative Commons Attribution 4.0 International license.

In addition to defining promoters, we were able to define ∼250 intrinsic terminators in Z. mobilis (Fig. 6). Their characteristics were remarkably similar to those found for E. coli intrinsic terminators (51), suggesting that the use of synthetic terminators vetted in E. coli (53) should work similarly in Z. mobilis. Although ρ-dependent termination remains to be characterized in Z. mobilis, our finding that only a third of the TTSs map to predicted intrinsic terminators suggests that ρ-dependent termination may be as important in Z. mobilis as it is in E. coli. This observation is of particular importance because improved solvent (e.g., ethanol) resistance is an important engineering goal for the development of Z. mobilis as a chassis microbe for biomass conversion to bioproducts, and ethanol activates ρ-dependent termination in E. coli through effects on both transcription and translation (59). An improved understanding of ρ-dependent termination in Z. mobilis will inform engineering efforts.

In conclusion, our multiomics analysis of Z. mobilis both improves the understanding of transcription and translation programs in this important alphaproteobacterium and provides new tools for its exploitation using synthetic biology approaches.

## MATERIALS AND METHODS

### Strains and growth media.

Zymomonas mobilis subsp. *mobilis* ZM4 ATCC 31821 was obtained from the American Type Culture Collection (ATCC). Rich medium with glucose (RMG) contained 10 g yeast extract, 2.6 g KH_2_PO_4_, 5 g K_2_HPO_4_, and 20 g glucose per liter. Minimal medium with glucose (MMG) contained 20 g glucose, 2.6 g KH_2_PO_4_, 5 g K_2_HPO_4_, 0.5 g NaCl, 1 g (NH_4_)_2_SO_4_, 0.2 g MgSO_4_·7H_2_O, 25 mg Na_2_MoO_4_·2H_2_O, 10 mg CaCl_2_·2H_2_O, 1 mg calcium pantothenate, 25 mg FeSO_4_·7H_2_O, and 20 g glucose per liter and was adjusted to pH 6.4 with HCl. Cell growth was monitored in real time by light scattering (apparent optical density [OD]) at 600 nm using a Beckman Coulter (Brea, CA) DU720 spectrophotometer. The extracellular glucose concentration was measured using a YSI (Yellow Springs, OH) 2700 biochemistry analyzer. Starter cultures of ZM4 were grown overnight in RMG in an anaerobic chamber and used to inoculate 3 liters of medium in bioreactors from Applikon Biotechnology (Foster City, CA). Anaerobic cultures were headspace sparged with a 95% N_2_–5% CO_2_ gas mix at a rate of 150 ml · min^−1^, and cells were stirred at 300 rpm. Aerobic cultures were liquid-phase sparged with atmospheric air at a rate of 700 ml · min^−1^ and stirred at 500 rpm. Multiomics samples were collected at 50% glucose depletion (mid-glucose-phase time point; ∼10 g glucose/liter remaining in the growth medium) and 1 h after glucose depletion (stationary-phase time point; no glucose remaining in the growth medium).

### RNA isolation and transcriptomic library construction.

RNA samples were collected by adding 10 ml of culture to 1.25 ml of an ice-cold ethanol-phenol stop solution (5% [vol/vol] H_2_O-saturated phenol, pH <7, in ethanol). Cell pellets were collected by centrifugation and stored at −80°C, and RNA was subsequently extracted using the hot-phenol method as described previously (60). DNase-treated total RNA was processed by the University of Wisconsin Biotechnology Center Gene Expression Center for rRNA subtraction by the Illumina RiboZero Bacteria kit and paired-end RNA-seq library generation using the Illumina TruSeq stranded total RNA library kit.

TSS-seq libraries were constructed using adaptations of previously reported methods (22, 23). RNA 5′-pyrophosphohydrolase (RppH) (catalog number M0356S; New England BioLabs) was used in place of tobacco acid pyrophosphatase for the pretreatment of total RNA. For RppH-treated libraries, 2.5 μg total RNA was incubated with 20 U RppH and 2 μl 10× reaction buffer in a final volume of 20 μl at 37°C for 2 h. RppH-untreated samples had 4 μl H_2_O in place of RppH. TSS-seq 5′ adapters contained 4-mer in-line barcodes; after 5′ adapter ligation, three RppH-treated and three RppH-untreated samples were pooled at equal masses prior to rRNA depletion with the Illumina RiboZero Bacteria kit. Termination sequencing libraries were prepared as described previously (23). Like TSS-seq libraries, term-seq libraries used 2.5 μg DNase-treated total RNA as the input and 3′ sequencing adapters with a 5-mer in-line barcode. After 3′ adapter ligation, six samples were pooled at equal masses prior to rRNA depletion with the Illumina RiboZero Bacteria kit.

### RppH *in vitro* activity assay.

Incorporation-radiolabeled 26-nt RNA with the sequence 5′-pppATGTAGTAAGGAGGTTGTATGGAAGA (PPP-A26) was generated by the *in vitro* transcription of a C-less template DNA template produced from pMK110 by PCR with primers 5′-CGTTAAATCTATCACCGCAAGGG and 5′-CAGTTCCCTACTCTCGCATG using 200 μM ATP, 200 μM UTP, and 10 μM [α-^32^P]GTP (10 Ci · mmol^−1^) under reaction conditions described previously (61). PPP-A26 was purified by acid phenol and ethanol precipitation. Radiolabeled PPP-A26 was added to a TSS-seq RppH reaction mixture as described above, and an incubation time course was performed at 37°C. As controls, radiolabeled PPP-A26 without total RNA was incubated with 20 U RppH or water at 37°C for 3 h. The PPP-A26 and P-A26 bands from this time course were resolved by electrophoresis in a denaturing 22.5% (wt/vol) (19:1 acrylamide-bisacrylamide) polyacrylamide gel (8 M urea, 44 mM Tris-borate [pH 8.3], 1.25 mM Na_2_EDTA) and visualized by imaging with a Typhoon phosphorimager (GE Healthcare) to monitor the conversion of PPP-A26 to P-A26 by RppH.

### TSS-seq data analysis and TSS identification.

TSS-seq libraries were sequenced at the University of Wisconsin Biotechnology Center DNA Sequencing Facility at 1 by 50 bp on the Illumina HiSeq 2500 system. In-line 5′ adapter barcodes were used to demultiplex libraries using fastx_barcode_splitter (fastx toolkit version 0.0.13.2) using –bol and default parameters. Barcodes were removed from the 5′ ends of reads and sequencing adapter readthrough was removed using Trimmomatic version 0.30 (62) with the following parameters: HEADCROP:6 ILLUMINACLIP:TruSeq3-SE.fa:2:30:10 MINLEN:25. Reads were aligned to the Z. mobilis ZM4 chromosome and plasmid sequences under GenBank accession numbers CP023715.1, CP023716.1, CP023717.1, CP023718.1, and CP023719.1 using Bowtie version 1.0.0 (63) with the following parameters: -S -m 1 –phred33-quals -v 2. Read 5′-only coverage was calculated for each position in the genome for both plus and minus strands using BEDTools (64) version 2-2.20.1 genomeCoverageBed with the following parameters: -5 -d.

Pearson correlation coefficients were calculated across all samples using genome-wide 5′-only read coverage values (referred to as nucleotide coverage here), and any biological replicates with a correlation coefficient of <0.9 were excluded from subsequent analyses. Nucleotide coverage data were prefiltered to remove positions with zero read coverage across biological replicates. The remaining positions were then filtered again to remove positions with a replicate-averaged coverage lower than the 95th-percentile value. For each condition, DESeq2 v1.14.1 (65) on R version 3.3.0 was then used to identify positions in RppH-treated samples with higher read coverage (i.e., setting altHypothesis = greater) than in RppH-untreated samples. DESeq2 was run in both paired and unpaired sample designs. TSSs were defined as positions with higher read coverage with an adjusted *P* value (FDR) of <0.05 in either the paired or unpaired DESeq2 tests or both.

In instances where multiple, adjacent positions (i.e., contiguous positions with no intervening base pairs) were identified as TSSs, the position with the highest RppH-treated read coverage (averaged across biological replicates) was selected as the final TSS, and the other adjacent position(s) was designated the secondary TSS(s). For σ^A^ promoter model building, TSSs from all six conditions were combined and further refined, first by removing secondary TSSs and then by calculating the number of conditions under which each TSS was identified. We then identified all instances of TSSs within 15 bp of each other and selected the TSS position that had been identified under the most conditions; this most common TSS was retained, and the remaining TSSs within 15 bp were removed (in cases of a tie, the upstream-most TSS was selected).

To validate TSSs identified by our method using published transcript 5′ ends or promoters, we aligned 5′-end coordinates identified by RACE (11, 12) and promoters identified by a reporter assay (33) with the Z. mobilis genome sequence under GenBank accession number CP023715.1. We then calculated the distance of known 5′ ends to the nearest TSS in our data set or the number of TSSs in our data set in each promoter region and compared these numbers to those found for a randomized set of TSSs in which the genome coordinates were rotated by 90° around the Z. mobilis genome (see Data Set S1 in the supplemental material). Both analyses revealed a highly significant association of our identified TSSs with the known transcript 5′ ends or promoter regions relative to the randomized data set (*P* < 0.0001 or 0.0004, respectively, by a Wilcoxon signed-rank sum test).

### Term-seq data analysis and TTS identification.

Term-seq libraries were sequenced at the University of Wisconsin Biotechnology Center DNA Sequencing Facility at 1 by 50 bp on the Illumina HiSeq 2500 system. In-line 3′ adapter barcodes were used to demultiplex libraries using fastx_barcode_splitter (fastx version 0.0.13.2) using –bol and default parameters. Barcodes were trimmed from the 5′ ends of reads and sequencing adapter readthrough was removed using Trimmomatic version 0.30 with the following parameters: HEADCROP:7 ILLUMINACLIP:TruSeq3-SE.fa:2:30:10 MINLEN:25. Reads were aligned to the Z. mobilis ZM4 chromosome and plasmid sequences (GenBank accession numbers CP023715.1, CP023716.1, CP023717.1, CP023718.1, and CP023719.1) using Bowtie version 1.0.0 with the following parameters: -S -m 1 –phred33-quals -v 2. Read 5′-only coverage was calculated for each position in the genome for both plus and minus strands using BEDTools version 2-2.20.1 genomeCoverageBed with the following parameters: -5 -d. Because term-seq libraries result in read sequences in the reverse complement to the starting RNA sequence, the strandedness of the read coverage data was reversed at the step of genomeCoverageBed such that the parameter “-strand-” was used to tabulate plus-strand read coverage and vice versa.

Pearson correlation coefficients were calculated across all samples using genome-wide 5′-only read coverage values (referred to as nucleotide coverage here), and any biological replicates with a correlation coefficient of <0.9 were excluded from subsequent analyses. For each sample, we identified TTSs using a custom script, run on R version 3.3.0, to perform a Poisson test on each position with a coverage value higher than the 95th-percentile value of all nonzero positions. For each position tested, a dynamic lambda value was estimated based on strand-specific genome-wide and sequence-wide (i.e., ZM4 chromosome, pZM3, and pZM33, etc.) average read counts as well as average read counts within 13-, 51-, 251-, 501-, and 1,001-bp windows centered on the tested position; the Poisson test was then performed with the largest dynamic lambda value using the R function ppois to calculate the probability of *X* ≥ *x*. The Benjamini-Hochberg method was applied for multiple-hypothesis testing correction, and sites with an FDR of <0.05 from each sample were selected. Positions identified in at least two biological replicates were then selected as preliminary 3′ termini for that condition. For the identification of processing sites and refinement of preliminary 3′ termini, the same method for preliminary 3′-terminus site identification was applied to all RppH-untreated TSS-seq samples to identify 5′-monophosphoryl RNA sites. All TSS positions were removed from this set of 5′-monophosphoryl sites, and the remaining positions were then compared with preliminary 3′-terminus positions. All instances of a 5′-monophosphoryl site directly downstream of a preliminary 3′ terminus (Fig. 2) were classified as processing sites. 3′ termini that overlapped tRNAs were also classified as processing sites. This analysis was performed for each condition in our experiment. Any combinations of 5′-monophosphoryl and preliminary 3′ termini that did not follow this convention (e.g., a 3′ terminus downstream instead of upstream of a 5′-monophosphoryl site) were removed. We reasoned that a 3′ terminus in one sample that had been identified as a processing site was most likely also a processing site in the remaining samples even if a corresponding 5′-monophosphoryl site was not found in the remaining samples. Therefore, we pooled processing sites identified across all conditions and cross-referenced this list against preliminary 3′ termini under each condition to derive a final set of TTSs by the removal of the processing sites.

### Prediction and analysis of intrinsic termination.

We predicted intrinsic terminators in the Z. mobilis chromosome using the software package TransTermHP (50) version 2.9 (available from transterm.ccb.jhu.edu) with default parameter settings but independent of genome annotations, yielding 1,746 predicted intrinsic terminators (Data Set S3). We scored a TTS as mapping to one of these intrinsic terminators if it occurred in positions 1 to 12 of the predicted terminator 3′-flanking sequence (i.e., within the predicted terminator 8-nt U tract plus the 4 nt downstream of the U tract), yielding 249 predicted intrinsic terminators to which one or more TTSs mapped (563 mapped TTSs) (Data Set S3). We noticed that some TTSs mapped to the 5′-flanking sequences of predicted terminators that were not predicted by TransTermHP as reverse complements to one of the 1,746 predicted terminators. Since these TTSs likely corresponded to termination in the flanking region of a bidirectional terminator that failed to score above the cutoff in TransTermHP, we added them to the list of TTSs that mapped to predicted terminators and appended an “r” to the listed terminator in Data Set S3. This consideration added 54 TTSs to the list of those mapping to predicted intrinsic terminators, for a total of 617 of 2,040 TTSs mapping to predicted terminators (Fig. 6A). To determine the predicted free energy of the formation of terminator hairpins (Fig. 6F), we used the DINAMelt server (66) with RNA version 3.0 energy rules at 37°C with 1 M NaCl. To obtain sequence logos (Fig. 6H, J, and K), we used the WebLogo server (67).

### RNA-seq and differential expression analysis.

RNA-seq libraries were sequenced at 2 by 126 bp on the Illumina HiSeq 2500 (v4) system at the University of Wisconsin Biotechnology Center DNA Sequencing Facility. Reads were filtered for low quality and adapter readthrough using Trimmomatic version 0.30 using the following parameters: ILLUMINACLIP:TruSeq3-PE.fa:2:22:10 SLIDINGWINDOW:4:28 MINLEN:75. Reads were aligned to the ZM4 chromosome and plasmid sequences (GenBank accession numbers CP023715.1, CP023716.1, CP023717.1, CP023718.1, and CP023719.1) using Bowtie 1.0.0 with the following parameters: -n 2 -l 25 -a -m 100. Gene read counts were obtained using the revised protein-coding annotations from this study, using RSEM version 1.2.3 (68) and Bowtie version 1.0.0 with the following parameters: –paired-end –calc-ci –estimate-rspd –forward-prob 0 –phred33-quals.

RSEM expected counts were used for downstream differential expression analysis. Pearson correlation of gene counts between biological replicates was used to detect outlier libraries. All the retained libraries had interreplicate Pearson correlation values of at least 0.95. Features representing rRNA and tRNA and genes with count sums of <5,000 across all remaining samples were removed from the matrix. Gene count normalization and differential expression testing were performed using DESeq2 version 1.14.1 run on R version 3.3.0.

### Constant-promoter catalog design.

Using the results of the above-described DESeq2 differential expression analysis, we defined all genes with a consistent expression level as those with log_2_-fold changes of more than −0.45 and less than 0.45 with an adjusted *P* value of >0.05 from each of the three pairwise comparisons between mid-glucose-phase samples. Genes with consistent expression across all three mid-glucose-phase samples (420 genes) were cross-referenced with the 2,675 TSS-gene pairs with an intergenic or leaderless TSS where the TSS was identified in all three mid-glucose-phase samples (637 TSSs and 385 genes), for a total of 94 candidate promoters. This preliminary list of candidate promoters was then manually inspected to identify TSS-gene pairs where the TSS is the upstream-most TSS for the candidate gene and where the TSS appeared to contribute to the majority of the expression of the gene.

### Ribosome-profiling library construction and data analysis.

Ribosome-profiling lysates were prepared as described previously (25). After isolation and polyacrylamide size selection of ribosome-protected footprints of between ∼30 and 35 nt, libraries were prepared as described previously (24). Libraries were sequenced at the Tufts University Core Facility at 1 by 51 bp on the Illumina NextSeq 550 system. The sequencing adapter was trimmed from reads using fastx_clipper (fastx-0.0.13.2) with the following parameters: -a CTGTAGGCACCATCAATATCTCGTATGCCGTCTTCTGCTTG -l 25 -v -Q33 -c. Trimmed reads were mapped to the Z. mobilis ZM4 chromosome and plasmid sequences (GenBank accession numbers CP023715.1, CP023716.1, CP023717.1, CP023718.1, and CP023719.1) using Bowtie version 1.0.0 with the following parameters: -S –phred33-quals -l 25 -k 1 –best.

### Proteogenomics analysis.

Protein sample processing and LC-MS/MS were performed as described previously (10). For proteogenomics analysis, a six-way translation of the Z. mobilis ZM4 chromosome and plasmid sequences was performed using MaxQuant 1.6.3.4 (26, 27) with the minimum amino acid sequence length (“Min. Length [AAs]”) set to 20 and using the bacterial and plant plastid translation table. Peptide spectra from all 19 samples were used to search a database built (using MaxQuant) from this six-way translation and default contaminant sequences using default parameters and the following user-specified parameters: variable modifications of acetyl (amino-terminal parameter called N-term), formyl-M (any N-term), Leu→Met (any N-term), and Val→Met (any N-term) and semispecific trypsin/P digestion. Peptide sequences from spectrum matches reported in peptides.txt and modificationSpecficPeptides.txt outputs were parsed and converted to the corresponding genomic locations.

### Gene revisions.

The intersection of proteogenomic peptide hits and protein-coding genes was assessed using BEDTools2-2.27.0 intersectBed, and the results were parsed with a custom Perl script to categorize peptides as intergenic, antisense, in-frame overlapping (with the protein-coding gene), and out-of-frame overlapping (with the protein-coding gene). All intergenic, antisense, out-of-frame, and partially overlapping in-frame peptide hits were compared against ZM4 protein-coding gene annotations in GenBank records under accession numbers CP023715.1, CP023716.1, CP023717.1, CP023718.1, and CP023719.1 as well as protein-coding gene annotations from the NCBI PGAP reannotation (accession numbers NZ_CP023715.1, NZ_CP023716.1, NZ_CP023717.1, NZ_CP023718.1, and NZ_CP023719.1). These results informed a strategy in which we compiled all differences between protein-coding gene annotations from these two sources (using BEDTools subtractBed) and looked for peptide hits specific to regions that differed between the two sets of annotations (largely pertaining to gene 5′ ends); peptide hits supporting gene start codon revisions are noted with “proteogenomics evidence” in Table S1. Ribo-seq was then used to examine protein-coding gene differences without peptide hits by calculating the average ribo-seq read coverage within the remaining regions that differed between sets of gene annotations; the same was done for gene differences with peptide hits, and the two distributions were compared. Six regions were identified with an average ribo-seq coverage greater than the mean ribo-seq coverage of peptide hit regions, which are noted as “ribosome-profiling evidence” in Table S1. The remaining regions without proteogenomics peptide hits or ribo-seq support were manually examined, and the longest version of a protein-coding gene was selected unless the shorter version had a methionine start codon and the longer version did not. We then examined any remaining antisense and intergenic proteogenomic peptide hits, which led to the identification of a previously unannotated gene on plasmid pZM36 that was assigned the locus tag pZM36x049. For the sake of completeness, we added all 49 PGAP-unique gene features to our revised gene annotations; we note that 4 of these gene features were pseudogenes and that another 4 features corresponded to noncoding products. Of the 41 protein-coding features uniquely identified by PGAP, 6 were validated by proteogenomic peptides, as noted in Table S1, and another 3 were validated by ribo-seq in the analysis described above for start coordinate revisions. After protein-coding gene revisions were complete, we changed the product designation of hypothetical proteins to “uncharacterized protein” if proteogenomic peptide hits supported the production of a protein product (155 changes, with 249 hypothetical proteins remaining unchanged). Finally, PGAP reannotation of the Z. mobilis ZM4 chromosome and plasmid sequences resulted in differences in the start sites of the 23S and 16S rRNAs relative to previous annotations. We used RNA-seq, TSS-seq, and term-seq to examine rRNA gene loci, which allowed the identification of putative rRNA gene primary transcripts for all three rRNA gene loci in addition to validating and refining 23S and 16S rRNA annotations, respectively.

The Z. mobilis ZM4 GenBank records under accession numbers CP023715.1, CP023716.1, CP023717.1, CP023718.1, and CP023719.1 have been updated to incorporate gene annotation revisions. We note that because there was no change to the underlying DNA sequence in these records, the accession and version numbers of these records will remain the same; however, revised genes can be identified by a difference in the protein_id version number and by the assignment of a new GI number.

### σ^70^/σ^A^ flexible DNA-binding modeling.

We replicated a pipeline described previously (43) to produce a flexible DNA-binding model for σ^70^/σ^A^ using custom Python and Perl scripts. Briefly, in our implementation of malign, we first generated a heuristic consensus sequence by picking five sequences at random, and explored the entire landscape of alignments, before picking the alignment of the five sequences that yielded the highest information content. We then used this heuristic consensus as a template to which we aligned each of the sequences in the alignments, adding each aligned sequence to the consensus upon alignment. Upon aligning all sequences once, we eliminated the initial heuristically generated consensus and iteratively continued shuffling each sequence. These passes continued until the improvements in information contents dropped below a certain threshold. Our malign algorithm was used to identify sequences with a −10 element from which a preliminary −35 motif was built and subsequently optimized with malign. Our implementation of multiscan then used this preliminary −35 motif to identify final −35 sites and introduced penalties for nonoptimal spacer lengths using a gap penalty calculated as described previously (43).

### σ^70^/σ^A^ multiple-sequence alignment.

Amino acid sequences for Z. mobilis, C. crescentus, *T. aquaticus*, B. subtilis, and M. tuberculosis σ^A^ and E. coli σ^70^ were obtained from UniProt. InterPro 77.0 was used, via the European Bioinformatics Institute (EMBL-EBI) website, to annotate protein domains from the amino acid sequences obtained from UniProt. Clustal Omega (1.2.4) was used, via the EMBL-EBI website (69), to align amino acid sequences annotated by InterPro for the RNA polymerase sigma 70 region 1.2 domain (InterPro identifier IPR009042; Pfam identifier PF00140) and the RNA polymerase sigma 70 region 2 domain (InterPro identifier IPR007627; Pfam identifier PF04542).

### Data availability.

RNA-seq, TSS-seq, term-seq, and ribosome-profiling raw and processed data are available through the National Center for Biotechnology Information Gene Expression Omnibus under accession number GSE139939. The mass spectrometry proteomics data have been deposited to the ProteomeXchange Consortium via the PRIDE (70) partner repository with the data set identifier PXD016962. Our σ^A^/σ^70^ promoter model pipeline and all associated scripts are available through the GitHub repository (https://github.com/jmvera255/Vera_2020_mSystems).
