# Supplementary figures and images for: Genome-Scale Transcription-Translation Mapping Reveals Features of Zymomonas mobilis Transcription Units and Promoters
Source: mSystems. 2020 Jul 21;5(4):e00250-20. doi: 10.1128/mSystems.00250-20 (PMC7566282; doi:10.1128/mSystems.00250-20)

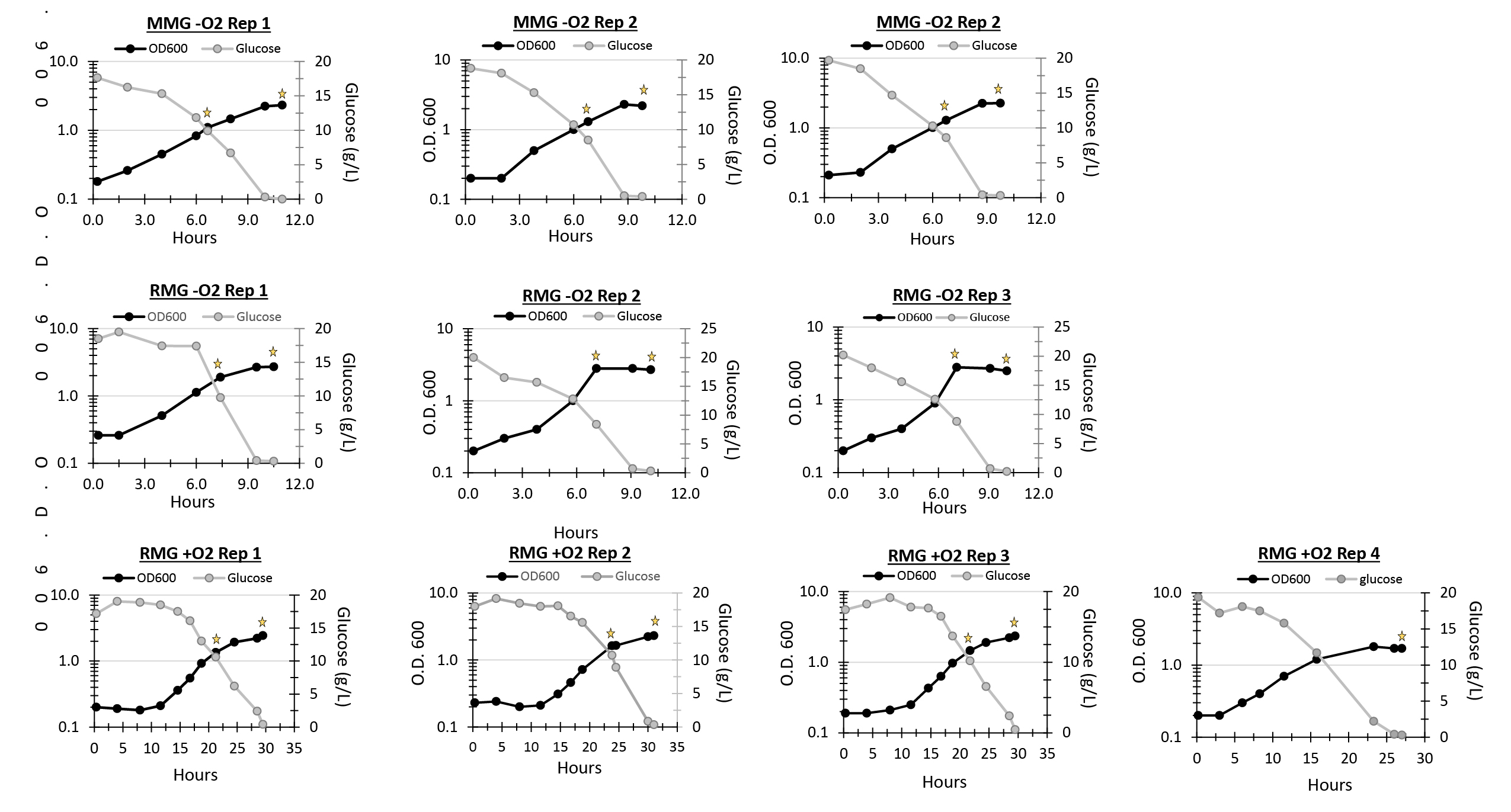

Supplement: FIG S1 [file mSystems.00250-20-sf001.jpg]

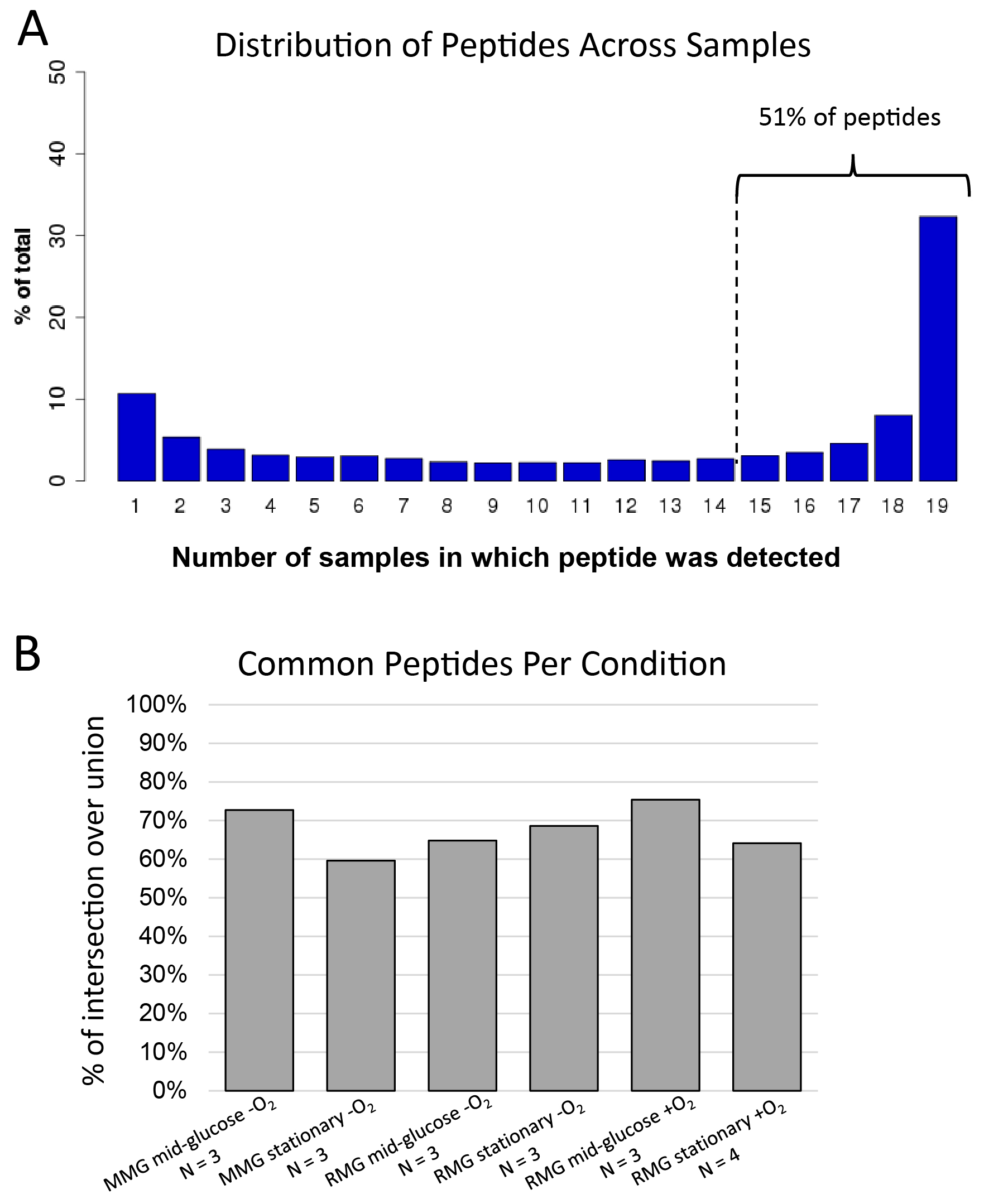

Supplement: FIG S2 [file mSystems.00250-20-sf002.jpg]

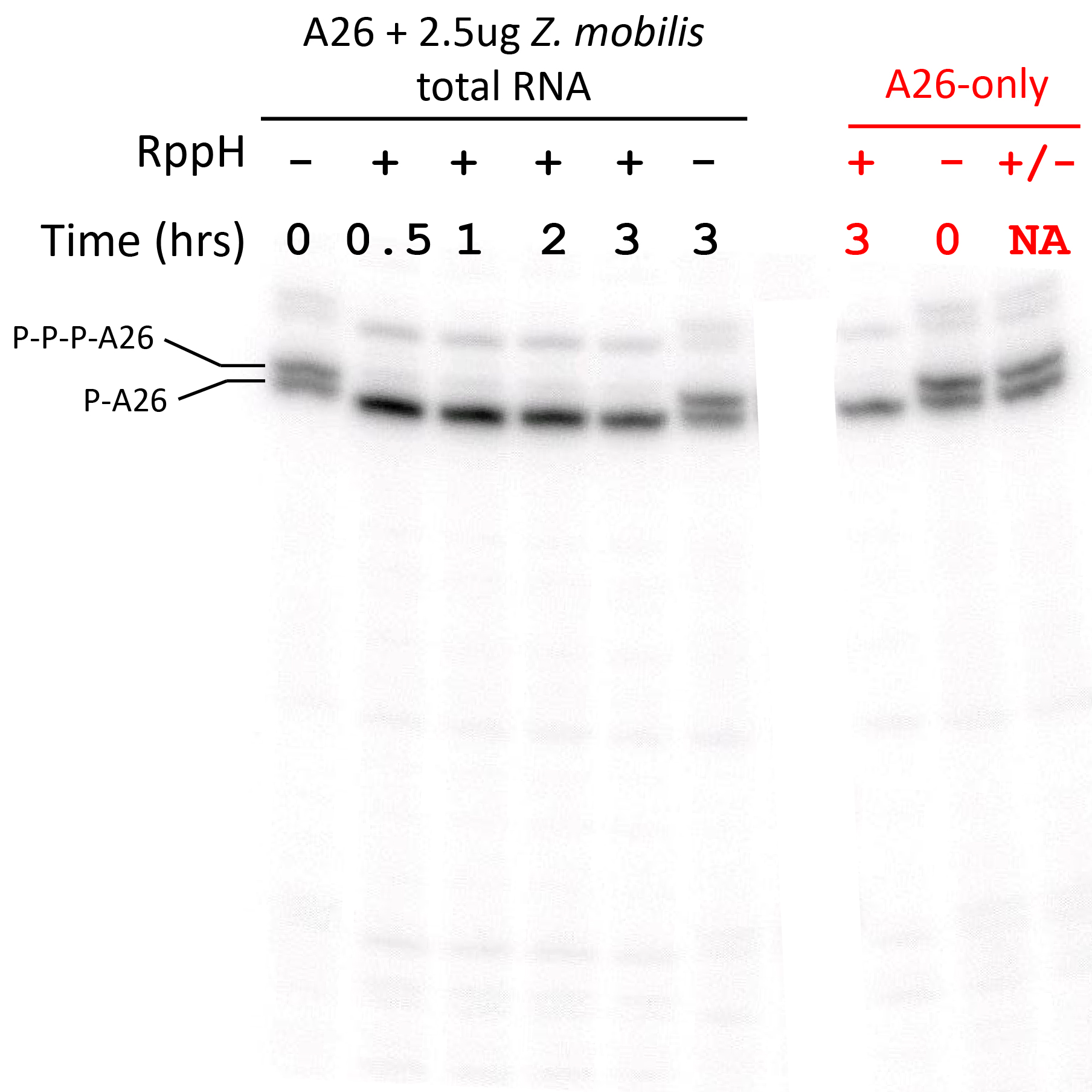

Supplement: FIG S3 [file mSystems.00250-20-sf003.jpg]

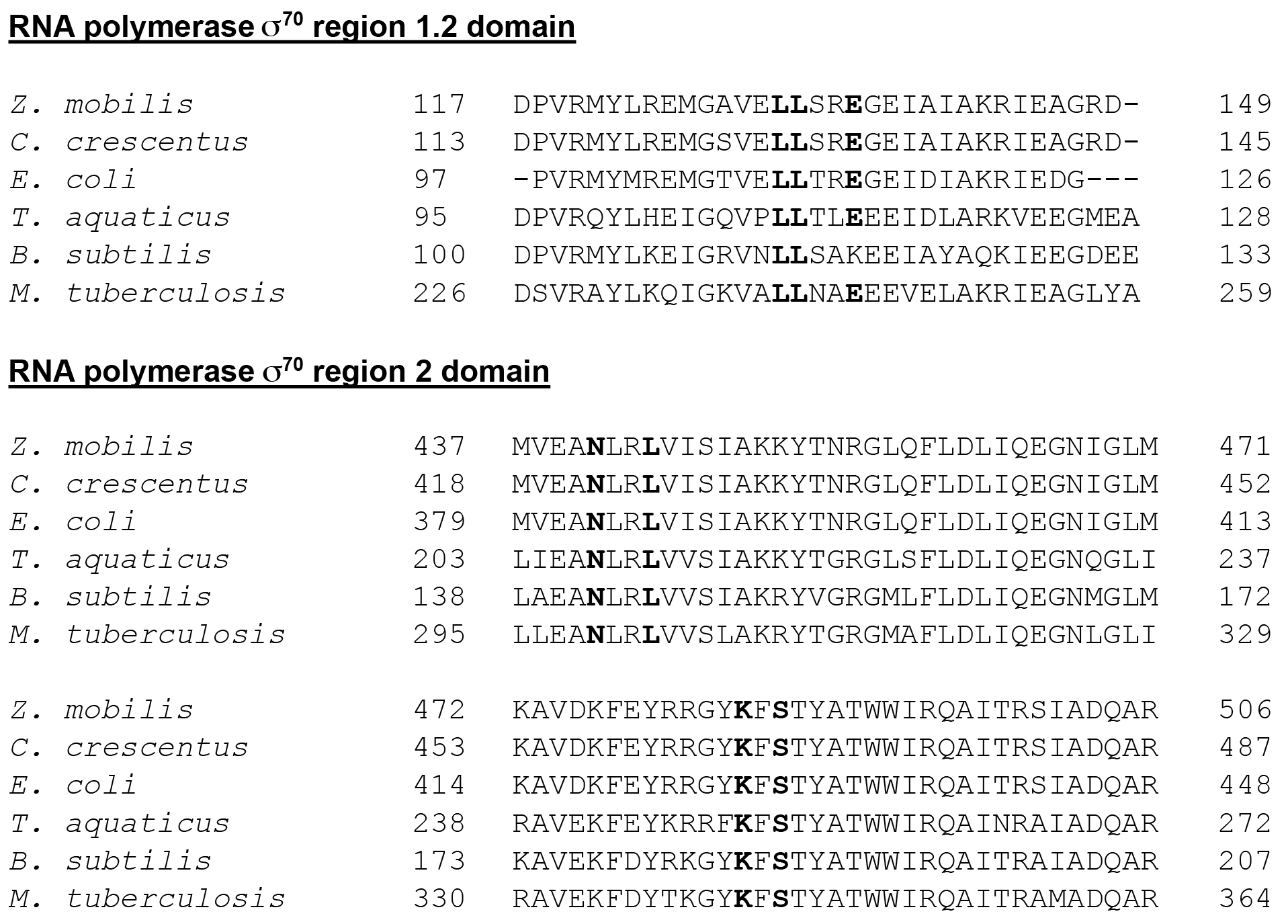

Supplement: FIG S4 [file mSystems.00250-20-sf004.jpg]
