# Supplementary material for: Genome-Scale Transcription-Translation Mapping Reveals Features of Zymomonas mobilis Transcription Units and Promoters
Source: mSystems. 2020 Jul 21;5(4):e00250-20. doi: 10.1128/mSystems.00250-20 (PMC7566282; doi:10.1128/mSystems.00250-20)
Supplement: TABLE S1 [file mSystems.00250-20-st001.pdf]

Table S1. List of revisions to Z. mobilis ZM4 gene annotations

Revision	Gene	Sequence	Start	Stop	Strand	Note
Predicted start change/longer gene	ZMO0031	ZM4	34635	37055	-	
Protein-coding genes added	ZMO2042	ZM4	86442	86648	+	old_locus_tag = ZMO1_RS00405
Predicted start change/longer gene	ZMO0124	ZM4	111117	112700	-	
Predicted start change/longer gene	ZMO0132	ZM4	120789	121607	+	
Predicted start change/longer gene	ZMO0145	ZM4	134055	135728	+	ribosome-profiling evidence
Protein-coding genes added	ZMO2043	ZM4	140799	141038	+	old_locus_tag = ZMO1_RS00645; proteogenomics evidence
Protein-coding genes added	ZMO2044	ZM4	154131	154574	+	old_locus_tag = ZMO1_RS00725
Protein-coding genes added	ZMO2045	ZM4	166334	166711	-	possible pseudogene
Predicted start change/shorter gene	ZMO0201	ZM4	195587	196171	-	
Protein-coding genes added	ZMO2046	ZM4	234003	234263	-	old_locus_tag = ZMO1_RS01040
Predicted start change/longer gene	ZMO0262	ZM4	263992	264639	+	
Protein-coding genes added	ZMO2047	ZM4	278860	279066	-	old_locus_tag = ZMO1_RS01200
Predicted start change/longer gene	ZMO0299	ZM4	303462	304715	-	proteogenomics evidence
Predicted start change/longer gene	ZMO0321	ZM4	320294	320791	+	proteogenomics evidence
Protein-coding genes added	ZMO2048	ZM4	330354	330536	-	old_locus_tag = ZMO1_RS01435
Predicted start change/longer gene	ZMO0363	ZM4	367280	368467	+	proteogenomics evidence
Predicted start change/longer gene	ZMO0375	ZM4	381469	382710	+	
Predicted start change/longer gene	ZMO0392	ZM4	395792	396295	+	
Protein-coding genes added	ZMO2049	ZM4	540759	540986	-	old_locus_tag = ZMO1_RS02425
New pseudogene assignment	ZMO2051	ZM4	603167	603948	+	ZMO1_RS02690 added from PGAP, assigned new name
Protein-coding genes added	ZMO2052	ZM4	685920	686111	+	old_locus_tag = ZMO1_RS03090
Protein-coding genes added	ZMO2053	ZM4	727374	727751	-	old_locus_tag = ZMO1_RS03280; proteogenomics evidence
Predicted start change/longer gene	ZMO0735	ZM4	737968	739326	-	proteogenomics evidence
Predicted start change/longer gene	ZMO0751	ZM4	750574	751017	+	
Protein-coding genes added	ZMO2054	ZM4	791565	791903	+	old_locus_tag = ZMO1_RS03545
Protein-coding genes added	ZMO2055	ZM4	807506	807703	-	old_locus_tag = ZMO1_RS03600
Predicted start change/longer gene	ZMO0811	ZM4	815680	816606	-	
Protein-coding genes added	ZMO2056	ZM4	819803	820003	-	old_locus_tag = ZMO1_RS03665; proteogenomics evidence
Predicted start change/longer gene	ZMO0836	ZM4	842402	843685	+	
Predicted start change/longer gene	ZMO0842	ZM4	848820	849998	+	
Protein-coding genes added	ZMO2077	ZM4	1063484	1063711	+	old_locus_tag = ZMO1_RS04730
New pseudogene assignment	ZMO2010	ZM4	1079960	1080251	+	replaced with ZMO1_RS04815 sequence
New pseudogene assignment	ZMO1083	ZM4	1090205	1092383	+	replaced with ZMO1_RS04890 sequence
Predicted start change/longer gene	ZMO1128	ZM4	1147103	1148263	+	
Predicted start change/longer gene	ZMO1138	ZM4	1157017	1157955	+	
Protein-coding genes added	ZMO2060	ZM4	1176607	1176954	+	old_locus_tag = ZMO1_RS05235
Predicted start change/longer gene	ZMO1179	ZM4	1199540	1200733	-	proteogenomics evidence
Predicted start change/longer gene	ZMO1202	ZM4	1228876	1229346	+	proteogenomics evidence
Protein-coding genes added	ZMO2061	ZM4	1230202	1230396	-	old_locus_tag = ZMO1_RS05455
Protein-coding genes added	ZMO2062	ZM4	1233613	1233939	-	old_locus_tag = ZMO1_RS05480
New pseudogene assignment	ZMO2063	ZM4	1268406	1268501	+	ZMO1_RS05645 added from PGAP, assigned new name
Protein-coding genes added	ZMO2064	ZM4	1312577	1312903	+	old_locus_tag = ZMO1_RS05855
Predicted start change/longer gene	ZMO1312	ZM4	1330256	1331671	+	ribosome-profiling evidence
Protein-coding genes added	ZMO2065	ZM4	1374915	1375160	+	old_locus_tag = ZMO1_RS06125
Protein-coding genes added	ZMO2066	ZM4	1377546	1377728	-	old_locus_tag = ZMO1_RS06140; ribosome-profiling evidence

Protein-coding genes added	ZMO2067	ZM4	1377718	1377975	-	old_locus_tag = ZMO1_RS06145; ribosome-profiling evidence
Protein-coding genes added	ZMO2068	ZM4	1378170	1378391	+	old_locus_tag = ZMO1_RS06150
Predicted start change/longer gene	ZMO1408	ZM4	1422199	1424418	-	
New pseudogene assignment	ZMO2069	ZM4	1431622	1431789	-	ZMO1_RS06400 added from PGAP, assigned new name
Protein-coding genes added	ZMO2070	ZM4	1468858	1468941	+	old_locus_tag = ZMO1_RS06550
Predicted start change/longer gene	ZMO1452	ZM4	1473741	1474970	-	
Predicted start change/longer gene	ZMO1456	ZM4	1476044	1476700	+	
Protein-coding genes added	ZMO2071	ZM4	1519387	1519569	-	old_locus_tag = ZMO1_RS06745
Protein-coding genes added	ZMO2072	ZM4	1531677	1531862	+	old_locus_tag = ZMO1_RS06810
Predicted start change/longer gene	ZMO1514	ZM4	1541878	1543002	-	
Protein-coding genes added	ZMO2073	ZM4	1604933	1605178	-	old_locus_tag = ZMO1_RS07085
16S rRNA 5' extention	ZMOr003	ZM4	1618912	1620401	-	
Predicted start change/shorter gene	ZMO1582	ZM4	1621373	1621864	-	
Protein-coding genes added	ZMO2074	ZM4	1635903	1636124	-	old_locus_tag = ZMO1_RS07225
Predicted start change/longer gene	ZMO1607	ZM4	1651528	1651896	-	ribosome-profiling evidence
Predicted start change/longer gene	ZMO1648	ZM4	1695624	1696361	+	proteogenomics evidence
Predicted start change/longer gene	ZMO1670	ZM4	1718174	1718605	+	
Predicted start change/longer gene	ZMO1702	ZM4	1753021	1755429	-	ribosome-profiling evidence
Predicted start change/longer gene	ZMO1772	ZM4	1817979	1819034	+	
16S rRNA 5' extention	ZMOr006	ZM4	1904061	1905550	-	
Predicted start change/longer gene	ZMO1863	ZM4	1911574	1912062	-	
16S rRNA 5' extention	ZMOr009	ZM4	1917913	1919402	-	
Predicted start change/longer gene	ZMO1884	ZM4	1937229	1937864	-	proteogenomics evidence
Predicted start change/longer gene	ZMO1892	ZM4	1945253	1945699	-	ribosome-profiling evidence
Protein-coding genes added	ZMO2075	ZM4	1945746	1945937	+	old_locus_tag = ZMO1_RS08660
Predicted start change/longer gene	ZMO1908	ZM4	1961633	1962127	-	ribosome-profiling evidence
Protein-coding genes added	ZMO2076	ZM4	1988876	1989217	-	old_locus_tag = ZMO1_RS08855
Predicted start change/shorter gene	ZMO2035	ZM4	1996574	1997365	+	
Predicted start change/longer gene	ZMO1971	ZM4	2010275	2011726	+	
New pseudogene assignment	ZMOp32x003	pZM32	4660	4975	+	replaced with ZMO1_RS09185 sequence
New pseudogene assignment	ZMOp32x017	pZM32	17610	18388	-	merged with ZMOp32x018
Protein-coding genes added	ZMOp32x030	pZM32	19567	19761	-	old_locus_tag = ZMO1_RS09255
Protein-coding genes added	ZMOp32x031	pZM32	19922	20131	-	old_locus_tag = ZMO1_RS09260
Protein-coding genes added	ZMOp32x032	pZM32	23758	24027	-	old_locus_tag = ZMO1_RS09285
Predicted start change/longer gene	ZMOp32x024	pZM32	24028	24453	-	
Protein-coding genes added	ZMOp32x033	pZM32	24447	24740	-	old_locus_tag = ZMO1_RS09295
Protein-coding genes added	ZMOp32x034	pZM32	28903	30363	+	old_locus_tag = ZMO1_RS09305; proteogenomics evidence
Predicted start change/longer gene	ZMOp32x028	pZM32	30360	31622	+	proteogenomics evidence
New pseudogene assignment	ZMOp33x024	pZM33	22216	23288	-	replaced with ZMO1_RS09435 sequence
Predicted start change/longer gene	ZMOp36x010	pZM36	7377	7913	+	
Predicted start change/longer gene	ZMOp36x015	pZM36	10613	13243	+	
Protein-coding genes added	ZMOp36x048	pZM36	15092	15454	-	old_locus_tag = ZMO1_RS09610; proteogenomics evidence
Protein-coding genes added	ZMOp36x049	pZM36	15559	15885	-	proteogenomics evidence
Protein-coding genes added	ZMOp36x050	pZM36	16240	16428	-	old_locus_tag = ZMO1_RS09620; ribosome-profiling evidence
Protein-coding genes added	ZMOp36x051	pZM36	16907	17233	-	old_locus_tag = ZMO1_RS09630; proteogenomics evidence
Protein-coding genes added	ZMOp36x052	pZM36	17338	17664	-	old_locus_tag = ZMO1_RS09635
Genes reassigned sequence	ZMOp36x022	pZM36	17693	18055	-	replaced with ZMO1_RS09640 sequence; proteogenomics evidence

Predicted start change/longer gene	ZMOp36x032	pZM36	23605	24075	-	proteogenomics evidence
Predicted start change/longer gene	ZMOp36x033	pZM36	24268	24579	+	
Protein-coding genes added	ZMOp36x053	pZM36	36440;1	36494;284	+	old_locus_tag = ZMO1_RS09510; gene spans end and start of plasmid sequence
Predicted start change/shorter gene	ZMOp39x003	pZM39	653	1141	+	
New pseudogene assignment	ZMOp39x019	pZM39	20461	20917	-	merged with ZMO1_RS09840
New pseudogene assignment	ZMOp39x029	pZM39	32862	33416	-	replaced with ZMO1_RS09885 sequence
New pseudogene assignment	ZMOp39x035	pZM39	38007	38324	-	ZMO1_RS09895 added from PGAP, assigned new name
Protein-coding genes added	ZMOp39x036	pZM39	38007	38324	-	old_locus_tag = ZMO1_RS09915
Protein-coding genes added	ZMOp39x037	pZM39	38314	38535	-	old_locus_tag = ZMO1_RS09920
